# Potential role of gut microbiota-LCA-INSR axis in high fat-diet-induced non-alcoholic fatty liver dysfunction: From perspective of radiation variation

**DOI:** 10.1016/j.crfs.2022.09.022

**Published:** 2022-09-27

**Authors:** Huiji Pan, Meiling Zhou, Zhao Ju, Jinhua Luo, Jing Jin, Liangfang Shen, Pingkun Zhou, Ruixue Huang

**Affiliations:** a Department of Occupational and Environmental Health, Xiangya School of Public Health, Central South University, Changsha, Hunan Province, 410078, China; b Department of Radiation Biology, Beijing Key Laboratory for Radiobiology, Beijing Institute of Radiation Medicine, AMMS, Beijing, China; c Department of Oncology, Xiangya Hospital, Central South University, China

**Keywords:** Gut microbiota, High fat diet, Radiation

## Abstract

Non-alcoholic fatty liver disease (NAFLD) is a progressive disease of the liver covering a range of conditions from hepatic steatosis to liver fibrosis. NAFLD could be induced by High-fat-diet(HFD). Ionizing radiation is widely used in medical diagnosis and therapy as well as is a common risk factor in occupational environment. Whether the exposure of various dose of radiation has effects on HFD-induced NAFLD remains unclear. Here, we reported that radiation exposure promoted HFD-induced NAFLD in a dose-response manner. Furthermore, the gut microbiota composition had significant difference among mice with or without radiation treatment. Specifically, the Bacteroidetes/Firmicutes ratio, the abundance of *A. muciniphila*, Butyricococcus, and Clostridiaceae decreased significantly in the mice with co-exposure of high dose of radiation and HFD treatment. A fecal transplantation trial (FMT) further verified the role of gut microbiota in the regulation of the liver response to co-exposure of high dose of radiation and HFD treatment. Notably, the gut microbiome analysis showed plasma lithocholic acid (LCA) level increased in the mice with co-exposure of high dose of radiation and HFD treatment. Following antibiotic and probiotic treatments there was a significantly decreased LCA bile acid concentration and subsequent promotion of INSR/PI3K/Akt insulin signaling in the liver tissues. Our results demonstrate that the co-exposure of radiation and HFD aggravates the HFD-induced NAFLD through gut microbiota-LCA-INSR axis. Probiotics supplementation is a potential way to protect against co-exposure of radiation and HFD-induced liver damage. Meanwhile, our study provide a new insight that population with potential HFD-induced damage should pay more attention on preventing from liver damage while exposing radiation.

## Highlights

• Gut microbiota-lithocholic acid-insulin receptor (LCA-INSR) axis involves the promotion effects of radiation on HFD-induced NAFLD**.**
• Probiotics improve the liver damage induced by co-exposure of radiation and HFD.

## Graphical abstract

**Figure:**
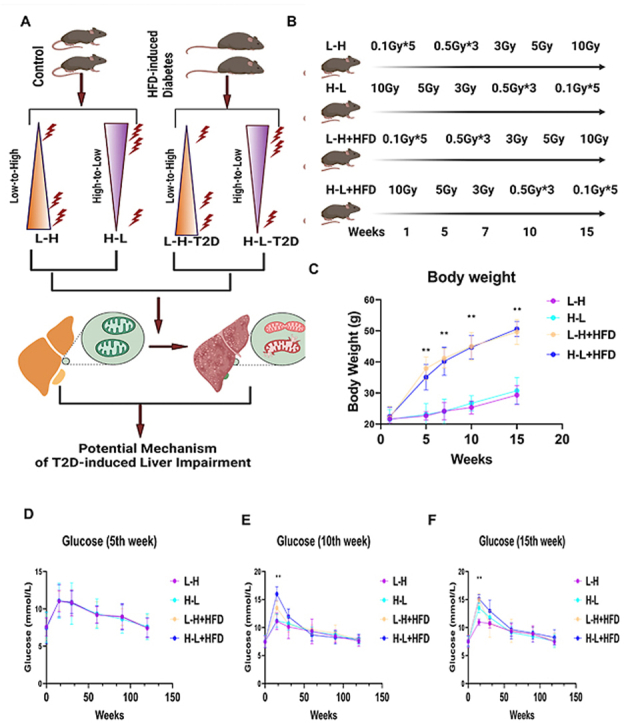

## 1 Introduction

Non-alcoholic fatty liver disease (NAFLD) is a progressive disease of the liver covering a range of conditions from hepatic steatosis to liver fibrosis (Moore et al., 2020). It is associated with a five-fold increased risk of incident diabetes in young adults (Chung et al., 2021). Approximately 30% of the general US population are affected by NAFLD, of whom 25% may develop further liver disease (Chalasani et al., 2018), while 25% of the European population suffer from NAFLD (Fazel et al., 2016). In China, which is the world's most populous country, the prevalence of NAFLD has surpassed the incidence in USA and Europe (Nan et al., 2021). Although many studies have revealed that lifestyle behavior can increase the NAFLD risk, the synergistic role of environmental and lifestyle factors in the development and progression of NAFLD should be considered, due to human exposure to multiple risk factors in the real world.

Ionizing radiation is a common environmental exposure factor, although it is not visible and cannot be touched. Many epidemiological and animal studies have suggested that radiation exposure is associated with an excess additive risk of secondary cancer (Thorby-Lister et al., 2018), inflammation disease (Yahyapour et al., 2018), and diabetes (Nylander et al., 2016; Mortazavi et al., 2016). There is evidence that ionizing radiation exposure can have multiple impacts on human health, that the hormesis and adaptive response to low dose radiation exposures (Vaiserman et al., 2021), DNA double-strand breaks (Huang and Zhou, 2021), and carcinogenesis induced by high doses of radiation (Huang and Zhou, 2020). Indeed, human expose various radiation dosage in environment or occupational exposure including Radiological therapy/examination, space flight (Cucinotta, 2022), nuclear power plant, accident of nuclear weapons, outdoor ultraviolet radiation or natural high background area such as Yangjiang, China (Cuiju et al., 2020). Over the past decades, although lots of studies of the effects of radiation have been published, most of them focused on the high dose or low dose, respectively. A study by W Yu et al. showed that mice pre-exposed to chronic low dose rate radiation are less susceptible to chromosome aberration induced by subsequent acute higher X-irradiation (Yu et al., 1995) indicating the dose exposure is a critical factor to influence human health. A study showed a dose-response relation between radiation exposure of pancreas and subsequent risk of diabetes (de Vathaire et al., 2012). Also, Therapeutic radiation exposure of the abdomen may increase the risk of adipose tissue dysfucntion (Huang et al., 2021). Importantly, as the fast food popular and HFD diet habit has been more and more public across the world, the obesity and diabetes incidences have been increasing largely during the past decades. Thus there may have larger amount of population encountered the chances of radiation including medical examination or radiotherapy. Furthermore, in some particular occupational environment including space flight, the astronauts may encounter various doses of radiation. Exposure of radiation from low dose to high dose may be in the astronauts from earth to the space, whereas exposure of radiation from high dose to low dose of radiation may be in the cancer patients with radiation therapy. Whether the effects of variation of radiation dosage on health remains unclear. In addition, the effects of an exposure to radiation in combination with lifestyle behavior, in particular, variation of radiation dosage in radiation exposure accompanied by most high-food diet (HFD) behavior, requires in-depth study. We therefore hypothesized that the effects of radiation on HFD-induced damage may be associated with the variation of radiation dosage.

There is emerging evidence that the bile acid-gut microbiota axis contributes to liver disease susceptibility (Shao et al., 2021), with the process being considered to be the language of an intricate molecular cross-talk between humans and their gut microbiota (Guzior and Quinn, 2021). Liver disease pathogenesis and progression could be changed by gut microbiome modification, which influences the bile acid pool (Hild et al., 2021). However, whether the bile acid-gut microbiota axis is involved in the synergistic effects of variation of radiation dosage and HFD patterns, and the potential molecular mechanisms, remains unknown.

Here, we found that the variation of radiation dosage of radiation alters the HFD-induced NAFLD progression. In particular, it was found that in HFD fed mice, exposure to radiation that gradually changed from a high to low dose (H-L) significantly promoted the progression of NAFLD through the regulation of bile acid via the gut microbiota-lithocholic acid-insulin receptor (IA-INSR) axis. Our findings explained how the co-exposure of variation of radiation dosage in radiation exposure and HFD altered the progression of NAFLD in mice, which improve our understanding of regulatory interactions between these two entities.

## 2 Results

### 2.1 The synergistic effect of the HFD and radiation exposure presented significantly different biological characteristics in mice

The western diet, which is a typical HFD, has been shown to induce NAFLD (Wan et al., 2021). In the real world, dietary behavior is often accompanied by exposure to multiple physical factors, in particular a varying range of natural radiation exposures. We designed an experiment to investigate the synergistic effects of a HFD and radiation exposure on liver function in mice. We treated mice with a radiation exposure that gradually changed from a low to high dose (L-H) or from a high to low dose (H-L) with or without a HFD (L-H + HFD, H-L + HFD) at abdominal region for 15 weeks (Figs. S1A–C, Figs. S2A–B). We found that, while there was no significant change in intestine tissues (Fig. S1D), there were significant changes in body weight (Fig. S2C) and glucose levels (Figs. S2D–F). There were no significant differences in glucose levels between the L-H and H-L groups, but mice in the H-L + HFD group had a higher glucose level compared to the L-H + HFD group and this trend increased over time. In addition, there were significant changes in liver function parameters, including the levels of liver aspartate alanine transaminase(ALT) (Fig. S3A), transaminase (AST)(Fig. S3B), liver total cholesterol(TC)(Fig. S3C), liver triglyceride (TG)(Fig. S3D), high density lipoprotein(HDL)(Fig. S3E), and low density lipoprotein (LDL)(Fig. S3F) among the four groups. An analysis of the liver tissue histology revealed that the lipid accumulation in the H-L + HFD group was greater than in the L-H + HFD, L-H, and H-L groups (Fig. S3G). Collectively, the characteristics of glucose levels and liver function were as follows: (i) no significant difference was observed between the L-H and H-L groups; (ii) the L-H + HFD group had a higher liver dysfunction than the L-H group; (iii) the H-L + HFD group had a higher liver dysfunction than the L-H group; (iv) the L-H + HFD group had a higher liver dysfunction than the H-L group; (v) the H-L + HFD group had a higher liver dysfunction than the H-L group; and (vi) the H-L + HFD group had a higher liver dysfunction than the L-H + HFD group. These results indicate that: (i) a HFD in synergy with radiation exposure triggered strong liver dysfunction; and (ii) while there were no differences in mice in relation to the variation of radiation dosage of radiation, mice fed with a HFD were more sensitive to the variation of radiation dosage of radiation.

### 2.2 The HFD enabled the liver to respond more sensitively to the spatial changes of radiation

**Fig. 1:**
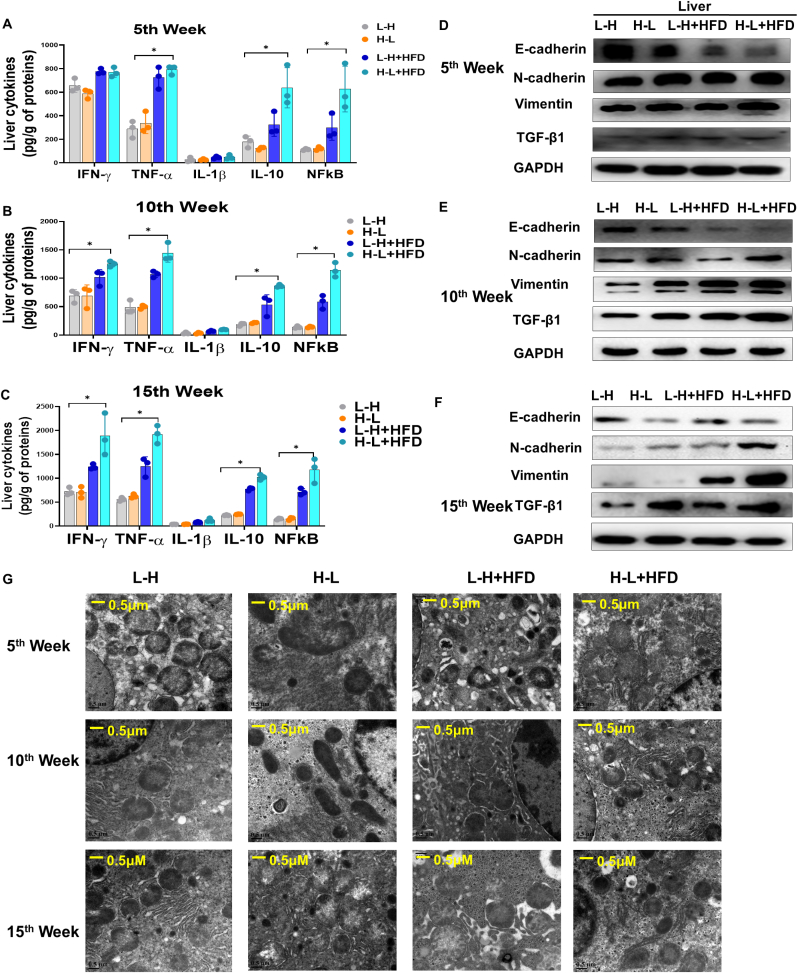
High-fat-diet synergized with repeated exposure to radiation variation from high dose to low dose triggers stronger liver fibrosis in mice. A. Liver cytokines levels, IFN-γ, TNF-a, IL-1β, IL-10 and NFkB in 4 groups at the 5th week. B. Liver cytokines levels, IFN-γ, TNF-a, IL-1β, IL-10 and NFkB in 4 groups at the 10th week. C. Liver cytokines levels, IFN-γ, TNF-a, IL-1β, IL-10 and NFkB in 4 groups at the 15th week. D. Western blot analysis of effects of co-exposure HFD and radiation variations on liver fibrosis-related proteins E-cadherin, N-cadherin, Vimentin, TGF-β1 expression at the 5th week post various intervention in mice. GAPDH subjected to the control reference. E. Western blot analysis of effects of co-exposure HFD and radiation variations on liver fibrosis-related proteins E-cadherin, N-cadherin, Vimentin, TGF-β1 expression at the 10th week post various intervention in mice. GAPDH subjected to the control reference. F. Western blot analysis of effects of co-exposure HFD and radiation variations on liver fibrosis-related proteins E-cadherin, N-cadherin, Vimentin, TGF-β1 expression at the 15th week post various intervention in mice. GAPDH subjected to the control reference. G. Representative electron microscopy images of liver tissues at indicated the 5th, 10th and 15th week among 4 groups, scale bar: 0.5 μM. Data are means ± standard deviation (SD). Mann-Whitney test or two-tailed unpaired Student's t-test were used for statistical analyses. *p < 0.05 indicates significant difference.

Considering the marked increase in liver dysfunction in the mice given a HFD diet accompanied by radiation exposure compared to those without the HFD, we determined the liver fibrosis-related inflammation factors and protein levels in mice. We found that mice in the H-L + HFD group exhibited a more severe activation and progressive inflammation response than the mice in the L-H + HFD, L-H, and H-L groups (Fig. 1A). The combination of the H-L and HFD treatments increased the expression of mice liver fibrosis-related biomarkers (Fig. 1B). The expression of both liver inflammation factors and fibrosis-related proteins increased in a time-dependent manner in response to the H-L variation of radiation dosage. A transmission electron microscopy (TEM) analysis further revealed that in the H-L + HFD group, the H-L and HFD treatment impaired the mitochondrial structure more severely than in the other three groups (Fig. 1C), with the ridge disappearing and appearing to have become fused and swollen. These results further indicated that the HFD made the liver more sensitive, with more inflammation and fibrosis in response to the H-L radiation exposure than in response to the L-H exposure. Figure D–F and Figs. S4A–C showed the EMT-related proteins expression at 5, 10 and 15 weeks.

**Fig. 2:**
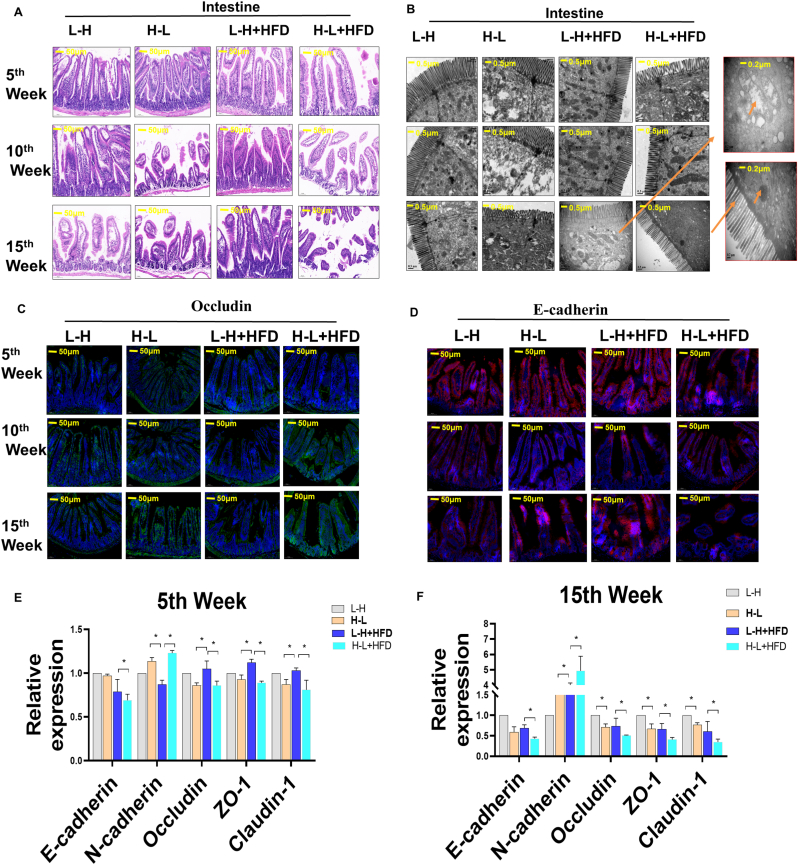
High-fat-diet synergized with repeated exposure to radiation variation from high dose to low dose triggers stronger intestinal barrier dysfunction and EMT progress. A. H&E staining of intestine of mice treated with radiation variants or combined with HFD at indicated the 5th, 10th and 15th week among 4 groups, scale bar: 100 μM. B. Representative electron microscopy images of liver tissues at indicated the 5th, 10th and 15th week among 4 groups, scale bar: 0.5 μM. C. Immunofluorescence detection for Occludin in mice treated with radiation variants or combined with HFD at indicated the 5th, 10th and 15th week among 4 groups, scale bar: 50 μM. D. Immunofluorescence detection for E-cadherin in mice treated with radiation variants or combined with HFD at indicated the 5th, 10th and 15th week among 4 groups, scale bar: 50 μM. E. Intestine EMT-related E-cadherin, N-cadherin relative expression and intestinal barrier tight junction-related Occludin, ZO-1 and Claudin-1 relative expression by qRT-PCR in mice treated with radiation variants with or without HFD fed at the 5th week. F. Intestine EMT-related E-cadherin, N-cadherin relative expression and intestinal barrier tight junction-related Occludin, ZO-1 and Claudin-1 relative expression by qRT-PCR in mice treated with radiation variants with or without HFD fed at the 15th week. Data are means ± standard deviation (SD). Mann-Whitney test or two-tailed unpaired Student's t-test were used for statistical analyses*p < 0.05 indicates significant difference.

Previous studies have reported that a HFD promotes multiple diseases by altering the gut microbiota, leading to changes in intestinal physiology (Mayneris-Perxachs et al., 2021). We hypothesized that increasing gut barrier damage due to the HFD was associated with the progression of H-L + HFD-induced liver inflammation activation and fibrosis. The gut intestinal barrier is vital for maintaining gut integrity and permeability to avoid endotoxemia, such as that caused by lipopolysaccharide (LPS) entry into the plasma and the subsequent low-grade inflammation, which has been considered to play a causal role in the development of non-communicable diseases (Huda et al., 2021; Huang et al., 2020a).The results showed that, compared to the other three groups, the H-L + HFD group exhibited more severe damage to the gut barrier, with broken membranes (Fig. 2A). The TEM analysis further showed that the mitochondrial structure was more severely altered (Fig. 2B) with the disappearance of the ridge, which appeared to be fused and swollen; lower expression of tight junction proteins, including Occludin, ZO-1, Claudi-1, and E-cadherin, and higher expression of fibrosis-related proteins, including N-cadherin, occurred (Fig. 2C–F). These results indicated the synergistic effect of the H-L radiation pattern and HFD, exacerbating gut barrier damage and inducing inflammation and fibrosis in the liver.

### 2.3 The H-L radiation pattern restructured the gut microbiota

**Fig. 3:**
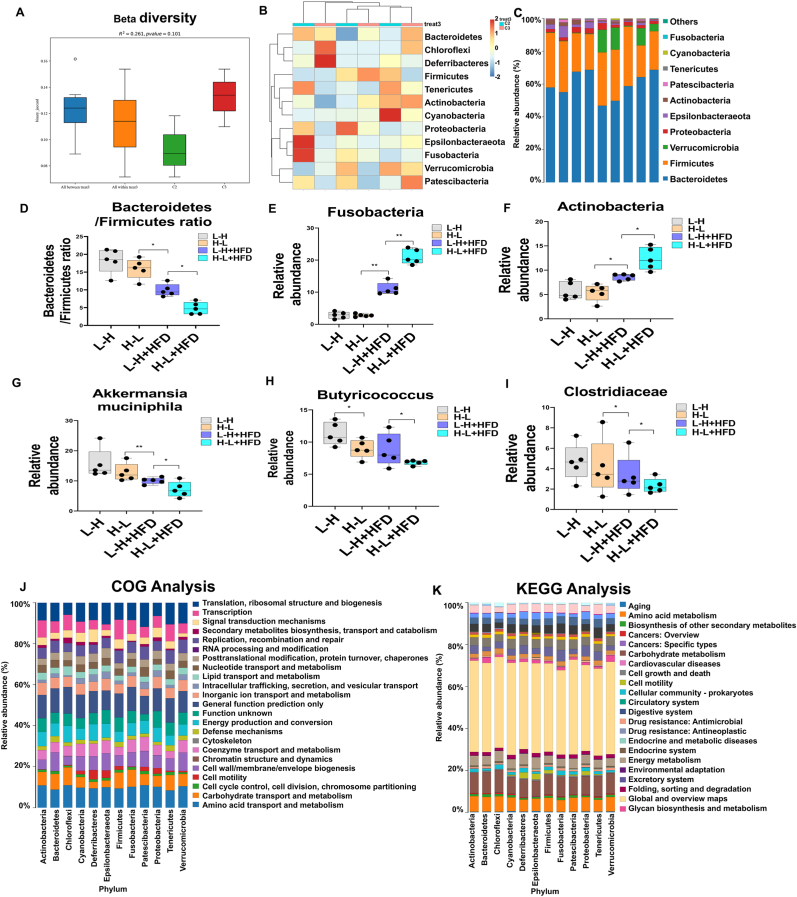
High-fat-diet synergized with repeated exposure to radiation variation from high dose to low dose induces significant impact on gut microbiome in mice. A. Analysis of Beta diversity among 4 groups used to compare the difference in gut microbiota composition among 4 groups by QIME software through unweighted unifrac method. B. Heatmap of gut microbiota abundance among 4 groups. Color represents abundance. Color from red to blue means relative abundance increased from high to low. C. Gut microbiota distribution columnar diagram among 4 groups. Various color represents various species. D. Bacteroidetes/Firmicutes ratio among 4 groups. E. Relative abundance of Fusobacteria among 4 groups. F. Relative abundance of Actlnobacterla among 4 groups. G. Relative abundance of Akkermansia mucinipila among 4 groups. H. Relative abundance of Butyrlcococcus among 4 groups. I. Relative abundance of Clostridiaceae among 4 groups.J. Picrust2 software is used to annotate the characteristic sequence to be predicted with the existing phylogenetic tree in the software, and img microbial genome data is used to output functional information, so as to speculate the functional gene composition in the sample, so as to analyse the functional differences between different groups (Luo et al., 2021). COG(Clusters of Orthologous Groups of proteins) analysis to predict the difference changed gut microbiota function. K. Potential KEGG (Kyoto Encyclopedia of Genes and Genomes) pathway analysis was used to predict changes in metabolic pathways of functional genes of microbial communities among samples in different groups. Data are means ± standard deviation (SD), n = 5 per group. Mann-Whitney test or two-tailed unpaired Student's t-test were used for statistical analyses. *p < 0.05 indicates significant difference.

The gut microbiota composition was further determined from fecal samples of the mice at 15 weeks using bacterial 16S rRNA gene v3-v4 amplicon sequencing as previously reported (Liu et al., 2019). The significant alterations in gut microbiota abundance at the phylum and species levels among the four groups are listed in Supplementary Tables 1 and 2, respectively. We found that mice in the H-L + HFD group had a significant difference in the beta diversity of gut microbiota abundance compared to the other three groups (Fig. 3A and B). Specifically, the Bacteroidetes/Firmicutes ratio, the abundance of *A. muciniphila*, Butyricococcus, and Clostridiaceae decreased significantly in the H-L + HFD group, while the abundance of Fusobacteria and Actinobacteria increased in the H-L + HFD group compared to the other three groups (Fig. 3D–I). A clusters of orthologous groups (COG) analysis of proteins predicted that the changed gut microbiota was mainly concentrated around lipid metabolism, signal transduction, and amino acid transport and metabolism (Fig. 3J). A Kyoto Encyclopedia of Genes and Genomes (KEGG) analysis showed that the altered gut microbiota groups were associated with infectious diseases, lipid metabolism, and amino acid metabolism (Fig. 3K).

To clarify further the role of gut microbiota in the regulation of the liver response to H-L radiation and HFD, we conducted a fecal transplantation trial (FMT) (Fig. S5A). As shown in Fig. S5B, mice receiving a fecal transplant from the H-L + HFD-treated group had higher glucose levels than the mice receiving a fecal transplant from the L-H + HFD and control groups. Mice receiving a fecal transplant from the H-L + HFD-treated group had more severe liver dysfunction, with significantly increased serum levels of ALT, AST, TC, TG, and LDL, but decreased levels of HDL (Figs. S5C–H). Hematoxylin and eosin (H&E) staining of the liver showed that mice receiving a fecal transplant from the H-L + HFD-treated group had a higher lipid accumulation in their liver and more severe gut barrier damage than mice receiving a fecal transplant from the L-H + HFD and control groups (Fig. S5I). We also found that the amount of liver inflammation cytokines increased, while the gut barrier tight junction-related proteins decreased in mice receiving a fecal transplant from H-L + HFD-treated mice compared to the other two groups (Figs. S5J–K).

To understand more fully the effects of gut microbiota-mediation on the gut barrier and liver function, we used an antibiotic cocktail to eliminate the gut microbiota of the mice, while probiotics were used as a beneficial positive control (Fig. S6A). We found that the plasma LPS level, and the serum hepatic-related TG and TC levels significantly decreased after the antibiotic and probiotic treatments (Figs. S6B–C). Furthermore, the relative expression of liver fibrosis-related genes and liver inflammation-related cytokines significantly decreased after the antibiotic and probiotic treatments (Figs. S6D–E), which indicated the critical role of gut microbiota. We conducted H&E staining, which revealed that the liver lipid accumulation was ameliorated. The degree of gut barrier damage was alleviated in the H-L + HFD group mice after the antibiotic and probiotic treatments (Fig. S6F), which indicated that the gut microbiota could mediate the progress of H-L + HFD-induced gut barrier damage and liver fibrosis. To explore whether the mitochondrial structure could be restored, we performed a TEM analysis. We found that mitochondrial numbers increased, with the fusion and swelling ameliorated after the antibiotic and probiotic treatments (Fig. S6G). These results demonstrated that the gut barrier damage and liver fibrosis induced by the synergistic effect of HFD and the H-L variation of radiation dosage of radiation were mainly mediated by modification of gut microbiota and gut metabolites. These effects could be ameliorated by antibiotic or probiotic treatments, and the plasma LPS level increased.

### 2.4 The insulin IRS-1/Akt/mTOR pathway was disrupted by the co-exposure to glucose and radiation

**Fig. 4:**
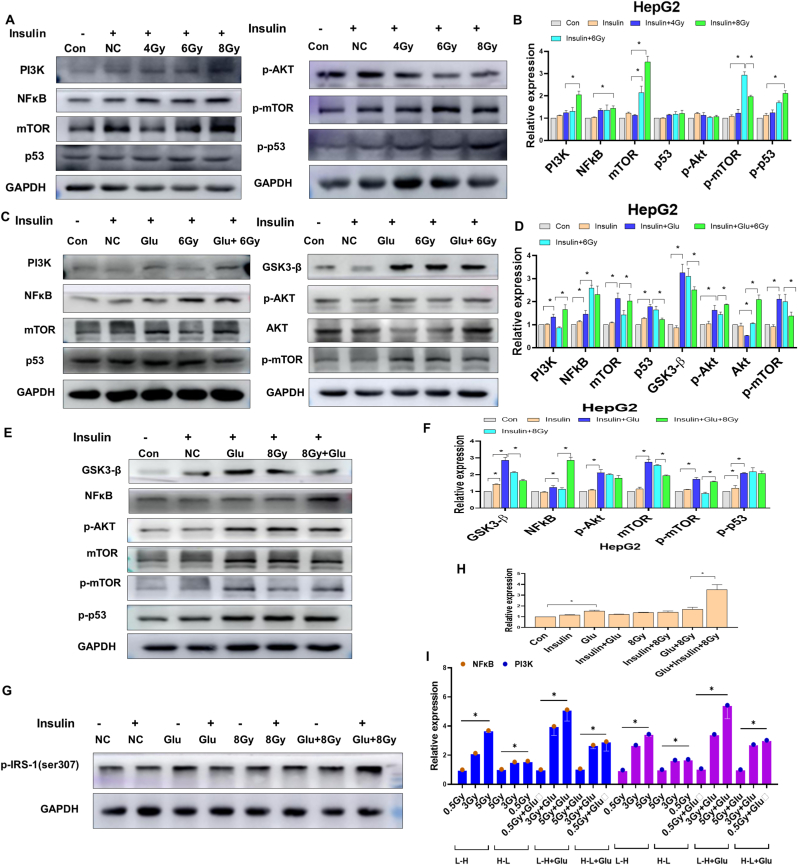
High glucose synergized with radiation treatment triggers insulin resistance in HepG2 cells. A. Representative of Western blot analysis of PI3K, NFkB, mTOR, p53, p-Akt, p-mTOR and p-p53 in liver cells treated with or without radiation. B. Quantitative of liver cell insulin resistance-related genes PI3K, NFkB, mTOR, p53, p-Akt, p-mTOR and p-p53 expression. C. Representative of Western blot analysis of PI3K, NFkB, mTOR, p53, p-Akt, p-mTOR and p-p53 in liver cells treated with or without high glucose combined with 6Gy radiation. D. Quantitative of liver cell insulin resistance-related genesPI3K, NFkB, mTOR, p53, p-Akt, p-mTOR and p-p53 expression with or without high glucose combined with 6Gy radiation. E. Representative of Western blot analysis of GSK3-β, NFkB, mTOR, p-Akt, mTOR, p-mTOR and p-p53 in liver cells treated with or without high glucose combined with 8Gy radiation. F. Quantitative of liver cell insulin resistance-related genes GSK3-β, NFkB, mTOR, p-Akt, mTOR, p-mTOR and p-p53 expression with or without high glucose combined with 8Gy radiation. G. Representative of Western blot analysis of p-IRS-1 in liver cells treated with or without high glucose combined with 8Gy radiation. H. Quantitative of liver cell insulin resistance-related genesp-IRS-1 expression with or without high glucose combined with 8Gy radiation. I. NFkB and PI3K mRNA expression were detected by qRT-PCR at indicated accumulation radiation exposure of various doses. Cells in L-H group were exposed 0.5Gy first and then exposed 3Gy radiation at 24 h, 5Gy radiation at 48h. Cells in H-L group were exposed 5Gy first and then exposed 3Gy radiation at 24 h, 0.5Gy radiation at 48h. Cells in L-H + Glu group were treated with glucose and exposed 0.5Gy first and then exposed 3Gy radiation at 24 h, 5Gy radiation at 48h. Cells in H-L + Glu group were treated with glucose and exposed 5Gy first and then exposed 3Gy radiation at 24 h, 0.5Gy radiation at 48h.

Because mitochondrial function is a hallmark of the insulin signaling pathway, we speculated that H-L + HFD-induced liver lipid accumulation may be caused by disruption to the insulin pathway. We then detected insulin resistance signaling using HepG2 cells by focusing on the IRS1/Akt/mTOR signaling pathway. In agreement with our hypothesis, we found that: (i) radiation exposure increased PI3K, NFkB, mTOR expression, but had no influence on p53 and p-p53 (Fig. 4A and B) while the effects of radiation on PI3K, NFkB, mTOR expression were dose-dependent; (ii) compared to the cells that received radiation, those that received glucose + radiation exhibited a higher expression of PI3K, NFkB, Akt, mTOR, and GSK3-β, but there were no alterations in the p53 expression (Fig. 4C and D), while the effects of glucose + radiation on NFkB, Akt, mTOR, and GSK3-β expression were dose-dependent(Fig. 4E and F); (iv) compared to the cells that received radiation, those that received glucose + radiation exhibited a higher expression of p-IRS-1(Fig. 4G and H). Further study showed that NFkB and PI3K expression increased with the accumulation of various doses of radiation and glucose (Fig. 4I).

To understand more fully the effects of glucose + radiation on the insulin signaling pathway, we used an antibiotic cocktail to eliminate the gut microbiota of the mice, with a probiotic treatment used as a beneficial positive control. We found that gut TLR4 and MYD88 expression decreased after the antibiotic or probiotic treatments in the H-L + HFD group (Fig. S7A). Immunohistochemistry (IHC) detection showed that E-cadherin and ZO-1 expression increased after the antibiotic or probiotic treatments in the H-L + HFD group (Fig. S7B). Furthermore, INSR, PI3K, Akt, and mTOR expression decreased after the antibiotic or probiotic treatments in the H-L + HFD group (Fig. S7C). We further found that in the HepG2 cells the protein expression of PI3K, Akt, and mTOR increased post radiation exposure, but decreased at a high radiation rose (8 Gy) compared with the increased expression at 4Gy. Consistent with the results of previous animal studies, the investigation of the cell insulin signaling pathway suggested that the H-L + HFD-induced liver fibrosis was mediated by gut microbiota through disruption of the gut barrier TLR4/MYD88 and liver IRS1/Akt/mTOR signaling pathways.

### 2.5 The H-L + HFD treatment promoted intestinal inflammation and liver dysfunction through a reduction in the levels of isocaproic acid (IA)

**Fig. 5:**
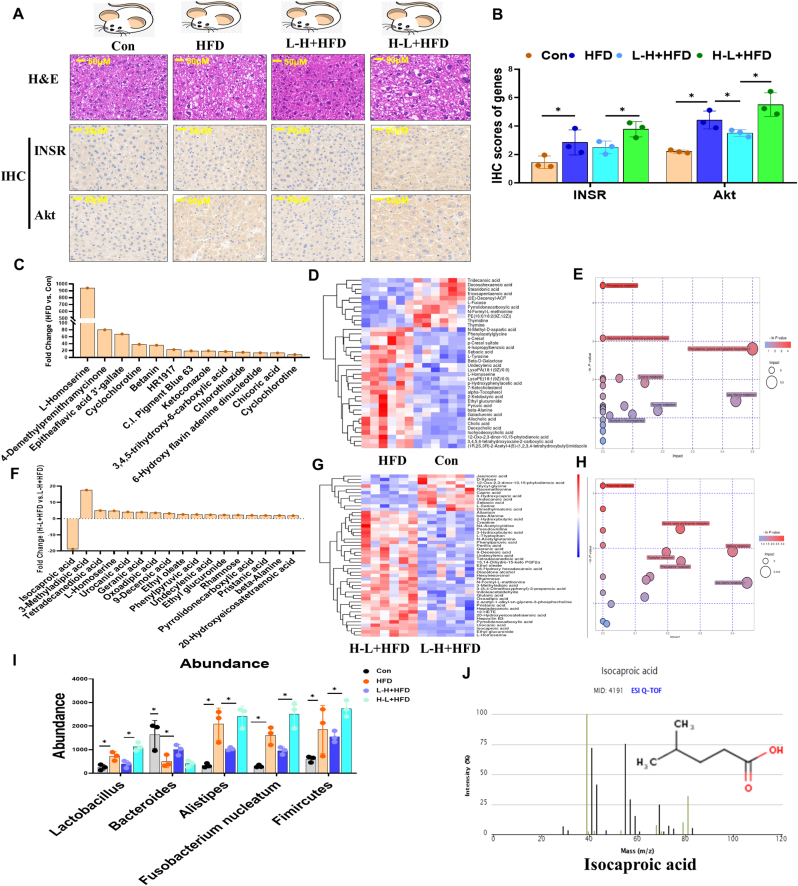
Profiling of plasma metabolomics under HFD synergizes L-H or H-L radiation setting. Mice were divided into four groups, con, HFD, L-H + HFD, H-L + HFD. Liver tissues were collected at 15th week post HFD synergizes L-H or H-L radiation setting. A. H&E staining of liver tissues in 4 groups, scale bar, 50 μM. Immunohistochemistry detection for INSR and Akt in liver tissues. Scale bar: 50 μM. B. Quantitative of INSR and Akt expression in liver tissues in 4 groups. C. Top 10 upregulated plasma metabolites in mice with HFD fed compared with normal control. D. Cluster Heatmap with metabolite properties between HFD and control groups, respectively in mice plasma metabolomics. E. Potential metabolism pathway analysis was conducted between HFD and control groups, respectively in mice plasma metabolomics. F. Top 10 upregulated plasma metabolites in mice with H-L synergized with HFD setting compared with L-H synergized with HFD setting. G. Cluster Heatmap with metabolite properties between H-L + HFD and L-H-HFD groups, respectively in mice plasma metabolomics. H. Potential metabolism pathway analysis was conducted between H-L + HFD and L-H-HFD groups, respectively in mice plasma metabolomics. I. Gut microbiota abundance among 4 groups. J. Isocaproic acid (IA) molecular structure and the OD value. Data are means ± standard deviation (SD). Mann-Whitney test or two-tailed unpaired Student's t-test were used for statistical analyses*p < 0.05 indicates significant difference.

To determine the mechanism underlying the alteration of the gut microbiota composition and its association with increased gut barrier damage and liver fibrosis risk, we compared the gut microbiome between the HFD vs. control and H-L + HFD vs. L-H + HFD using high-performance liquid chromatography (HPLC). Fig. 5A and B shows the INSR and Akt expression were increased to a greater extent in the H-L + HFD group than in the L-H + HFD group. Compared with the HFD group, the levels of L-homoserine, 4-demethylpremithramycinone, and epiteaflavic acid 3′-gallate increased significantly in the HFD group (Fig. 5C and D). These changed metabolites were mainly concentrated in the beta-alanine metabolism, phenylalanine, tyrosine, and tryptophan biosynthesis pathways (Fig. 5E). Compared with the L-H + HFD group, the IA level decreased significantly, but the levels of 3-methyladipic acid, tetradecanedioic acid, and L-homoserine increased in the H-L + HFD group (Fig. 5F and G). Consistent with previous animal and cell studies, these changed metabolites were mainly concentrated in the glucose metabolism-related pathways, e.g., glycine, serine, and threonine metabolism (Fig. 5H). Fig. 5I showed the alteration of various bacteria species among four groups. Fig. 5J showed the Isocaproic acid (IA) molecular structure.

**Fig. 6:**
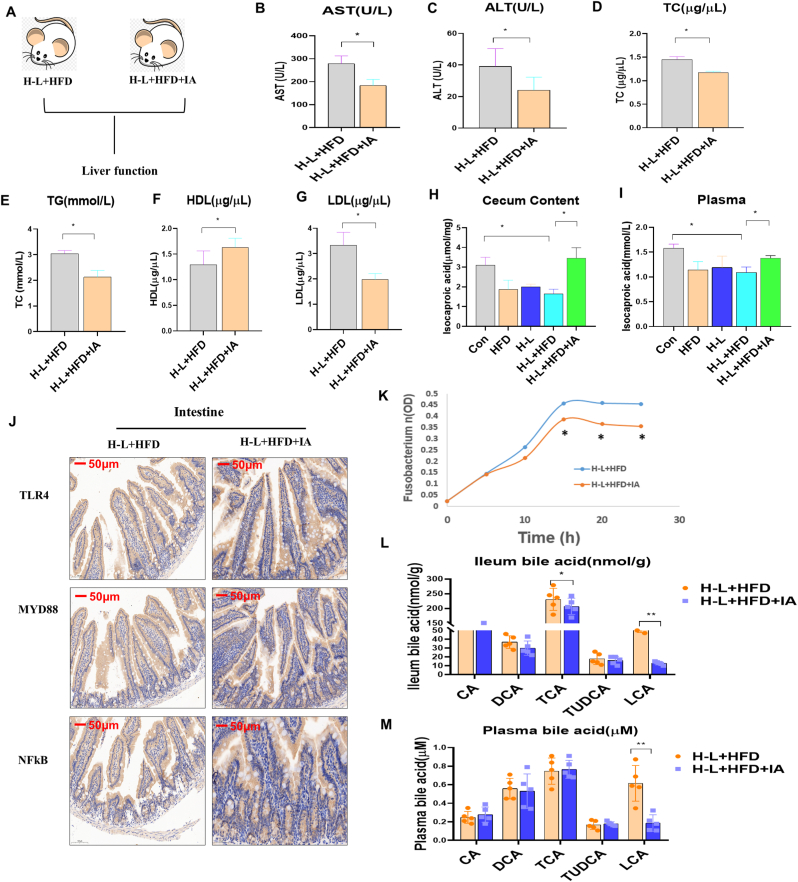
IA attenuated H-L + HFD-induced liver dysfunction in mice. Mice were divided into 2 groups, H-L + HFD, H-L + HFD + IA. Liver tissues were collected at 5th week post HFD synergizes H-L radiation or IA setting. A. Timeline of mice subjected to H-L + HFD, H-L + HFD + IA. B. AST level. C. ALT level. D. TC level. E. TG level. F. HDL level. G. LDL level. H. IA level in cecum content. I. IA level in plasma. J. Immunohistochemistry detection for TLR4, MYD88 and NFkB in intestinal tissues. Scale bar: 50 μm. K. Growth curve of F. nucleatum in cecal extracts (0.2 mg/ml) from 2 groups (3 technical replicate of 6 biological replicates for each group). L. ileum bile acid, CA, DCA, TCA, TUDCA and LCA levels in 2 groups. M. Plasma bile acid, CA, DCA, TCA, TUDCA and LCA levels in 2 groups. Data are means ± standard deviation (SD). Mann-Whitney test or two-tailed unpaired Student's t-test were used for statistical analyses*p < 0.05 indicates significant difference.

To explore the role of IA in H-L + HFD-induced liver function damage and intestinal inflammation, we compared the differences between the H-L + HFD and H-L + HFD + IA groups (Fig. 6A). Compared with the H-L + HFD group, the levels of AST, ALT, TC, TG, and LDL decreased after IA treatment, but the HDL level increased (Fig. 6B–G). We found that IA levels in the cecum extract and plasma decreased in the H-L + HFD group compared with the H-L or HFD groups (Fig. 6H and I). We found that gut TLR4, MYD88, and NFkB expression decreased after IA treatment in the H-L + HFD group following an IHC determination (Fig. 6J). We further found that *Fusobacterium nucleatum* had a slower growth rate and lower maximum growth concentration in the cecal extract from mice after the IA treatment in the H-L + HFD group than in mice without the IA treatment (Fig. 6K), indicating that the increase in *F. nucleatum* abundance in H-L + HFD mice may have been due to the alteration of complex gut microbiota-produced metabolites. We then investigated the alteration of bile acid in H-L + HFD mice with or without an IA treatment. We found that two components of bile acid, ileum taurocholic acid (TCA) and lithocholic acid (LCA), decreased after the IA treatment (Figure 6L). Plasma LCA also decreased, but the levels of CA, DCA, and TUDCA were not influenced by IA (Figure 6M). These results were consistent with a previous report that the levels of LCA increased in patients with inflammatory bowel disease associated with an increased abundance of gut *F. nucleatum* (Weng et al., 2019), indicating that IA influenced H-L + HFD-induced liver function damage.

### 2.6 Lithocholic acid promoted H-L + HFD-induced liver dysfunction through the inhibition of INSR signaling

**Fig. 7:**
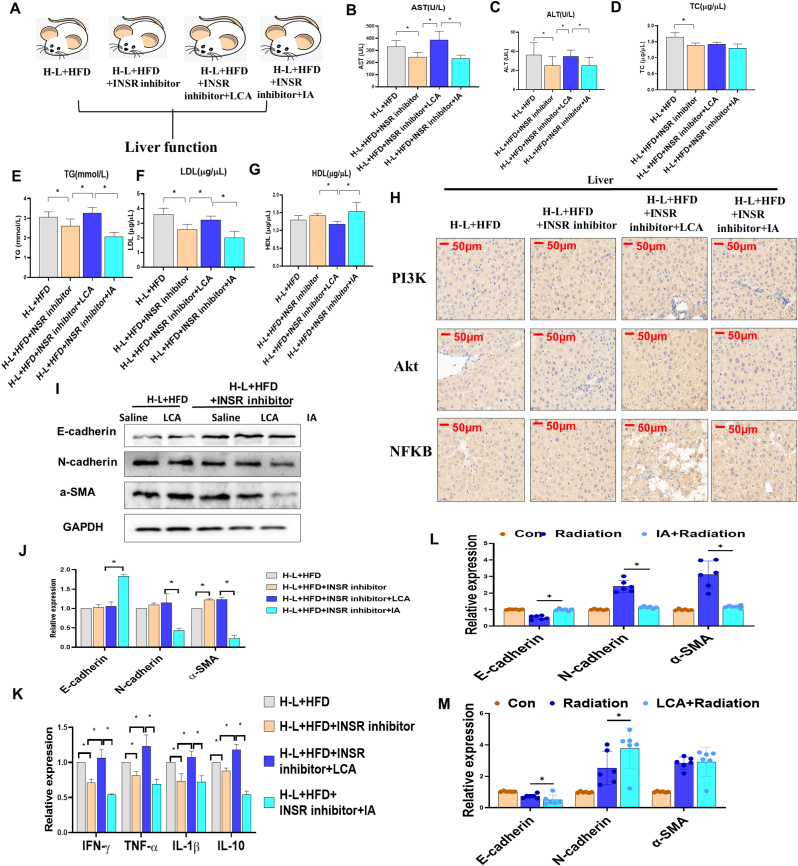
LCA inhibited H-L-HFD-induced liver fibrosis through regulating insulin resistance signaling pathway. Mice were divided into 4 groups, H-L + HFD, H-L + HFD + INSR inhibitor, H-L + HFD + INSR inhibitor + LCA and H-L + HFD + INSR inhibitor + IA. Liver tissues were collected at 5th week post HFD synergizes H-L radiation or LCA or IA setting. A. Timeline of mice subjected to H-L + HFD, H-L + HFD + INSR inhibitor, H-L + HFD + INSR inhibitor + LCA and H-L + HFD + INSR inhibitor + IA. B. AST level. C. ALT level. D. TC level. E. TG level. F. LDL level. G. HDL level. H. Immunohistochemistry detection for PI3K, Akt and NFkB in liver tissues. Scale bar:50 μm. I. Western blot analysis (and J. quantification) of effect of LCA (50 mg/kg) on liver fibrosis-related proteins, E-cadherin, N-cadherin a-SMA expression in mice. K. Quantification of liver inflammation-related mRNA expression. L. E-cadherin, N-cadherin and a-SMA mRNA expression in mice liver tissues with or without radiation and IA treatment were detected by qRT-PCR. M. E-cadherin, N-cadherin and a-SMA mRNA expression in mice lung tissues with or without radiation and IA treatment were detected by qRT-PCR. Data are means ± standard deviation (SD). Mann-Whitney test or two-tailed unpaired Student's t-test were used for statistical analyses*p < 0.05 indicates significant difference.

Previous reports have indicated that LCA is a secondary bile acid and an increase in its concentration is associated with accelerated insulitis, resulting in an increased risk of diabetes (McGlinchey et al., 2020). We therefore investigated the potential mechanism underlying LCA-mediated liver dysfunction (Fig. 7A). Because insulin signaling dysregulation is a hallmark of HFD-induced liver lipid accumulation through the critical receptor INSR (Dongiovanni et al., 2017), we used INSR inhibitor linsitinib (Meijles et al., 2021) to inhibit INSR signaling. We found that serum levels of AST, ALT, TC, TG, and LDL increased, but the HDL level decreased after an LCA treatment in the H-L + HFD + INSR inhibitor group (Fig. 7B–G). Liver PI3K and Akt expression decreased, but NFkB expression increased after an LCA treatment in the H-L + HFD + INSR inhibitor group (Fig. 7H), indicating that LCA decreased the expression of INSR signaling downstream genes, resulting in the inhibition of INSR signaling and promoting the progression of liver dysfunction. This result was consistent with a previous report that inhibition of the INSR/PI3K/Akt signaling pathway leads to glucose/insulin-stimulated hepatic stellate cell activation and extracellular matrix (ECM) production (Zhang et al., 2014). We further used Western blotting to test the expression of liver fibrosis-related biomarkers, and found that the inhibition of INSR expression decreased E-cadherin expression, but increased N-cadherin and a-SMA expression (Fig. 7I and J). The relative expression of liver inflammation factors increased after LCA treatment in the H-L + HFD + INSR inhibitor group (Fig. 7K). The relative expression of E-cadherin decreased after radiation but increased after IA treatment, whereas N-cadherin and a-SMA increased after radiation but decreased after IA treatment (Figure 7L). LCA treatment had the opposite effects (Figure 7M). These observations indicated that IA may against the radiation-induced fibrosis but LCA may promote the radiation damage. Meanwhile, LCA may exert an acceleration effect on H-L + HFD-induced liver fibrosis by targeting liver INSR signaling.

## 3 Discussion

Radiation exposure has a number of complex outcomes, including beneficial and non-beneficial roles in health, but the mechanisms involved remain unclear. A number of studies have shown that a HFD is an NAFLD risk due to the alteration of the gut microbiota composition (Mu et al., 2021). Recent studies have shown that cancer patients with obesity have a higher radiotherapy resistance in clinical settings (Sabol et al., 2020), indicating that a HFD-related life style is not only a risk factor associated with many diseases but may also play a role in the development of diseases combined with other environmental factors. Radiation exposure, a common environmental physical factor known for its double-edged ability to provide beneficial or non-beneficial outcomes, is used in medical diagnosis or therapy and can improve the liver immune function at low radiation doses (Zhang et al., 2021a). However, there is also a risk of oxidative liver damage after frequent high doses of radiotherapy (Sharma et al., 2021). The molecular pathogenic mechanisms of radiation-induced liver injury remain unclear. Previous reports have indicated it associates with vascular changes, increased collagen synthesis, sequential activation of key growth factors and cytokines including tumor necrosis factor alpha (TNF-α), transforming growth factor beta (TGF-β), as well as early effects of DNA damage, oxidative stress and reactive oxygen species production which resulting in the hepatocellular apoptosis and inflammatory reaction. In addition, the activation of compensatory proliferation of myofibroblastic is a critical cellular event for underlying hepatic fibrosis (Kim and Jung, 2017).

In this study, through a comparison of treatments with different variation of radiation dosage exposures, and combined with a HFD diet, we identified the specific mechanism by which a varying pattern of radiation exposure and a HFD alters the intestinal microbiota, damages the intestinal barrier, and increases LCA levels, thus promoting the development and progression of HFD-induced NAFLD via the activation of liver INSR/PI3K/Akt signaling. Depleting the gut microbiota by antibiotic treatment and the inhibition of INSR signaling eventually ameliorated HFD-induced liver damage via the LCA-INSR/PI3K/Akt axis.

*Fusobacterium nucleatum*, which is generally found in human dental plaque and is one of the most abundant oral commensal flora, is associated with various human diseases, including lung disease and periodontal infection after entering the blood circulatory system (Stokowa-Soltys et al., 2021). Studies have shown that this bacteria can migrate from its original oral site to other parts of body and cause diseases such as cancer. However, *F. nucleatum* also has an anti-inflammatory role through the production of butyric acid (Stokowa-Soltys et al., 2021). Asal Milhen et al. found that mice fed a HFD diet had an increased *F. nucleatum* abundance, with increased body weight and glucose tolerance (Milhem et al., 2021). Another study found a causal link between the activation of the LPS pathway by *F. nucleatum* and HFD-induced inflammation (Blasco-Baque et al., 2012). Consistent with these findings, we found that an increase in *F. nucleatum* abundance led to an increase in the LCA level and further promoted NAFLD through the activation of liver INSR signaling.

We identified IA as the specific metabolite that inhibited the growth of *F. nucleatum*, which explained the inhibition of *F. nucleatum* in antibiotic or probiotic-treated mice. There is emerging evidence that IA, which is one of the short-chain fatty acids and the product of the anaerobic intestinal bacterial fermentation of dietary fiber (Zietek et al., 2021), is positively associated with the body mass index(BMI) (Irving et al., 2016) and hepatic lipid metabolism (Zietek et al., 2021). Obesity in pregnancy can reduce the amount of IA in the stool and a decrease in the IA level would also reduce liver function (Honkoop et al., 1997). Consistent with previous reports, our results further indicated that the reduction of IA levels can be caused by the co-exposure of long term radiation exposure and a HFD.

Bile acids, a class of amphipathic steroids produced in the liver and extensively modified by the gut microbiota, are critical actors in the onset and progression of NAFLD (Li et al., 2021). Bile acids can act as signaling molecules, regulating liver cellular homeostasis through interaction with the Farnesoid X receptor(FXR), resulting in liver lipid metabolism (Radun and Trauner, 2021). The reported effects of bile acids on NAFLD make it an attractive therapeutic target in HFD-induced liver diseases. Masahiro M et al. developed an ileal bile acid transporter inhibitor and found that it improved HFD-induced hepatic steatosis by ameliorating gut microbiota dysbiosis in NAFLD model mice (Matsui et al., 2021). The liver is the central organ in lipid metabolism and studies have found that bile acids can activate a series of signaling pathways, such as the protein kinase B (AKT) signaling pathway that regulates lipid metabolism (Kwong et al., 2015). Lithocholic acid is a secondary bile acid, with high hydrophobicity and cytotoxicity, that is transformed by gut bacteria from a primary bile acid, causing more cell wall damage than the primary bile acids (Chiang, 2017). Lithocholic acid has been found to promote carcinogenesis (Ma et al., 2018) in the liver in animal models. A population-based epidemiological study showed that high LCA levels were strongly associated with advanced liver fibrosis (Kwan et al., 2020). Liver injuries induced by LCA treatment include the impacts on signaling pathways associated with inflammation and the proliferation of G protein-coupled receptor (TGR5)-dependent signaling (Klindt et al., 2019). Genetic polymorphisms related to insulin resistance will increase NAFLD susceptibility (Nobakht et al., 2020). Consistent with previous reports, we found that LCA promoted H-L + HFD-induced liver dysfunction through the activation of the INSR signaling pathway. These findings indicate that LCA might be the direct cause of HFD-induced NAFLD in the context of a radiation exposure varying from a high to low dose, with radiation likely to be a concomitant factor.

INSR/IRS/PI3K/Akt is classical insulin pathway in the process of glucose metabolism. Many studies have found that inhibition of this pathway due to environmental toxicants, e.g., bisphenol A, can activate an inflammatory response and lead to NAFLD (Lin et al., 2019). Yang P et al. found that the use of liraglutide could ameliorate NAFLD in diabetic mice through the activation of the IRS/PI3K/Akt signaling pathway (Yang et al., 2019). Zhang F et al. found the use of tetramethylpyrazine could reduce glucose and the insulin-induced activation of hepatic stellate cells through inhibition of the INSR-mediated PI3K/Akt pathway (Zhang et al., 2014). Consistent with these reports, our results showed that an increased LCA level in plasma could inhibit the INSR/PI3K/Akt pathway, leading to the development and progression of combined HFD and radiation-induced NAFLD. This further confirmed that the insulin pathway in NAFLD induced by environmental radiation exposure and a HFD can be disrupted. Considering that the INSR/PI3K/Akt pathway is a well-clarified pathway in insulin resistance, we suggest that future studies should investigate the underlying mechanism of how the INSR/PI3K/Akt pathway plays a role in which NAFLD is induced by a combination of environmental physical factors and lifestyle factors.

Limitations about this study should be mentioned. First, the radiation exposure levels in this study are remaining high, caution is required while interpreting these findings. Second, collagen levels in the liver and liver sections stained with Sirius red to reflect the degree of fibrosis and the effect of no experimental radiation exposure should be considered in the further study. Third, population study including cohort study or case-control study should be considered in the future to evaluation the effects of various radiation doses on human health.

## 4 Conclusions

In summary, we determined that variation of radiation dosage of radiation exposure altered the HFD-fed gut microbiota composition in mice through the regulation of gut barrier tight junction-related protein expression, leading to lower IA levels and increased LCA levels, and eventually the inhibition of the liver insulin INSR/PI3K/Akt pathway, thus promoting NAFLD development and progression. These findings have improved our understanding of the molecular mechanisms by which the gut microbiome can be regulated by a combination of environmental and lifestyle factors, and provides a novel strategy for the prevention or treatment of HFD-induced NAFLD. However, coupled with the over-physiologic doses of radiation used in this study, caution is required while interpreting these findings to human accidental/occupational/environmental radiation exposure.

## 5 Methods and materials

### 5.1 Mice, radiation, chemicals and cell lines

Male C57BL/6 mice were purchased at 8 weeks of age from the Hunan SJA Laboratory Animal Co., LTD, China. All of the mice were housed at the Animal Laboratory Division, Xiangya School of Public Health, Central South University, China. All animal procedures and testing were conducted according to the National Legislation and local guidelines of the Laboratory Animals Center at the Central South University. The study and research protocols were approved by the Institutional Animal Review Board of Central South University (2020sydw0110). In addition, all animals in the study were treated humanely with regard to the alleviation of suffering. All animal procedure experiments were approved by the Committee on Animal Use and Care of Central South University, China. All the mice were maintained in a specific-pathogen-free (SPF) environment with controlled conditions of a 12 h light/dark cycle at 20–22 °C and 45 ± 5% humidity. After 1 week of acclimation, the mice were used for the study with the group design detailed below.

To investigate the effects of spatial-temporal of radiation and synergization with HFD on liver metabolic impairments, mice were divided into four groups: L-H group, H-L group, L-H + HFD group and H-L + HFD group. The mice in the L-H group and H-L were fed ad libitum with a conventional diet. The mice in the L-H + HFD group and the H-L + HFD were exposed to different spectrum of radiation accompanied by HFD fed. The ingredients of high fat diet contained 24.15% fat, 22.51% protein and 38.34% carbohydrate purchased from XIETONG Bio. Company, Nanjing, China. For L-H treatment, the dosage spectrum was 0.1Gy of radiation administered to mice 5 times at the first 5 weeks, then 0.5Gy of radiation administered to mice 3 times within the 5th week and 7th week, 3Gy was administered at abdomen part at the 7th week, 5Gy was administered at abdomen part at the 10th week, 10Gy at abdomen part at the 15th week at room temperature, whereas for H-L treatment, the dosage spectrum was 10Gy of radiation administered to mice 1 time at the first week, then 5Gy of radiation administered to mice at abdomen part 1 time at the 5th week and 3 Gy of radiation administered to mice at abdomen part 1 time at the 7th week, 0.5Gy was administered at the 10th week 3 times, 0.1Gy was administered at the 15th week 5 times at room temperature. Cells and animals were subjected to irradiation treatment for 24 h with a dose range 0–10 Gy delivered with the Varian linear accelerator (2100EX, San Francisco, California, USA). Body weight, food intake and water consumption were recorded. The mice were killed at the 5th, 10th and 15th weeks to collect serum and tissues for further detection of various parameters. The radiation source was provided by the Department of Oncology, Xiangya Hospital, Central South University (CSU), Changsha, 410008, People's Republic of China (Zhang et al., 2021b).

Lithocholic acid (LCA) was purchased from Sigma (CAS No. 434-13-9). Linsitinib (HY-10191) were ordered from MedChem Express, MCE and dissolved in 25 mM tartaric acid). Isocaproic acid (IA) was purchased from Sigma (CAS No. 646-07-1). D-glucose was purchased from Solarbio (G8150). The antibodies used for Western blot analysis targeted the following proteins: GAPDH (CST, 5174), E-cadherin (CST, 14472), PI3K (Abcam, ab73262), Akt (Proteintech, 10176-2-AP), p-Akt (Abcam, ab81283), IRS-1 (CST, 2382), p-IRS-1 (CST, 2381), mTOR (Bioworld, bs1844), p-mTOR (CST, 5536S), PYCR1 (Abcam, ab279385), p53 (CST, 2527S), p-PI3K (CST, 4228T), Occludin (Abcam, ab216327), ZO-1 (Abcam, ab221547), Claudin-1 (Abcam, ab211737), N-cadherin (CST, 31593), INSR (CST, 23413).

The HepG2 cells were purchased from the Cell Bank of Type Culture Collection of the Chinese Academy of Sciences (Shanghai, China). Due to the previous reports that HepG2 was used in many metabolic studies (35893906, 35931467), we thus selected HepG2 in our study. The HepG2 cells were cultured in Dulbecco's modified Eagle's medium (DMEM; HyClone, Utah, USA) with 10% foetal bovine serum (FBS) and 1% antibiotic in a 5% CO2 cell incubator at 37 °C as described in our previous reports (Dai et al., 2020).

### 5.2 Antibiotic/probiotic treatment and fecal microbiota transplantation (FMT)

An antibiotic cocktail including ampicillin sodium salt (CA2031-5G), polymyxin B sulfate salt (CP8711-100 MG) and streptomycin sulfate (CS 10481-5G) was purchased from Coolaber Science & Technology. The probiotics included *Lactobacillus rhamnosus* (No. CICC, 20259) and *Lactobacillus reuteri* (No. CICC 6118), which were purchased from the China Center of Industrial Culture Collection (http://www.china-cicc.org/). Insulin was purchased from Coolaber Science & Technology (CI6561), China.

For investigation of the gut microbiota effects, mice were divided into four groups: L-H group, H-L group, L-H + HFD group and H-L + HFD group (in which mice were co-exposed to L-H or H-L radiation with HFD fed for 15 weeks). The mice fecal samples were collected at the 15th week and shipped to Biotree, Co., LTD, Shanghai, China for gut microbiome detection using 16rS technical. For investigation of the gut microbiota-dependent effects, mice were divided into four groups: Con, H-L + HFD, H-L + HFD + Antibiotics and H-L + HFD + Probiotics. In the H-L + HFD + antibiotics group (in which H-L + HFD + HFD mice were treated with an antibiotic cocktail that included 50 g/L ampicillin sodium, 100 U/L streptomycin, and 50 mg/L polymyxin B sulfate, as previously described (Rozos et al., 2018)) and the H-L + HFD + HFD + probiotics group (in which mice were treated with probiotics with dosages chosen based on our previous report (Ju et al., 2020)). Briefly, probiotics, including *Lactobacillus rhamnosus* (No. CICC, 20259) and *Lactobacillus reuteri* (No. CICC 6118), were purchased from the China Center of Industrial Culture Collection (http://www.china-cicc.org/). The probiotics were administered to the mice orally after being dissolved in PBS solution containing 50 × 10^9^ CFU of probiotics. The mice were administered antibiotics and probiotics prior to H-L + HFD co-exposure for 2 weeks, and then the experiment was performed for 10 weeks. The mice were killed at 10 weeks to collect serum and tissues for further detection of various parameters.

For the FMT study, the microbiota donors were mice treated with L-H + HFD or H-L + HFD for 10 weeks. Faeces of the donors were collected and dispersed in sterile Ringer working buffer, and the supernatant was mixed with skimmed milk for transplantation. Four-week-old germ-free male C57BL/6J mice were randomly divided into three groups(with 10 mice in each group), housed in sterile plastic package isolators and fed a sterilized normal diet. After 2 weeks of acclimation, the germ-free mice were orally gavaged with faecal suspensions from mice fed a HFD or subjected to L-H + HFD or H-L + HFD feeding. The mice were killed to collect tissues for further detection of various parameters at the end of the experiment. After all these experiments, the mice were fasted overnight before being euthanized. Blood samples were collected and kept at room temperature for 1 h to ensure complete clotting before centrifugation at 4 °C and 5000 rpm for 5 min to obtain serum samples. Faeces and tissues such as liver tissue, BAT, hippocampal tissue, and intestinal tissue were carefully collected, flash-frozen in liquid nitrogen and then stored at −80 °C until assessment.

To investigate the effects of IA, mice were divided into two groups: the H-L + HFD group, the H-L + HFD + IA group (in which mice were co-exposed to H-L radiation, HFD fed and fed IA). The mice were treated for 10 weeks and then killed to collected tissues for detection of various parameters.

To investigate the effects of LCA, mice were divided into four groups: the H-L + HFD group, the H-L + HFD + Linsitinib group (in which mice were co-exposed to H-L radiation, HFD fed and Linsitinib), the H-L + HFD + Linsitinib + LCA group (in which mice were co-exposed to H-L radiation, HFD fed and Linsitinib and LCA), the H-L + HFD + Linsitinib + IA group (in which mice were co-exposed to H-L radiation, HFD fed and Linsitinib and IA),. The mice were treated for 10 weeks and then killed to collected tissues for detection of various parameters.

### 5.3 Analysis of liver function parameters, bile acid measurement and growth curves of F. nucleatum

The insulin concentration was analysed with an enzyme-linked immunosorbent assay (ELISA) kit (Xinle Bio Co., Ltd., Shanghai, China) according to the manufacturer's instructions. For physiological function-related parameter detection, blood samples were collected in heparin-coated tubevia eye punctures and centrifuged, and then the plasma samples were stored at −80 °C. Plasma cholesterol, HDL, LDL, AST and ALT levels were measured with a biochemistry platform using an Olympus AU400 Chemistry Analyser provided by Central South University. Blood glucose concentrations were measured with a blood glucose test meter, and blood samples were obtained from the mice tails at 0, 15, 30, 60, and 90min after intraperitoneal injection of glucose (1.5 g/kg body weight).

For isocaproic acid assay, a 0.5 g fecal sample was suspended in a tube containing 5 mL of water and mixed intensively for 5 min. Using 5 M HCl solution, the pH of suspension was adjusted to 2–3. The samples were then shaken for 10 min and centrifuged for 20 min at 5.000 rpm. Subsequently, the supernatant was filtered (Ø 400 μm) and assayed using used an HPLC–MS gas chromatograph system (Shimadzu Corporation, Japan) (Skonieczna-Zydecka et al., 2018).

Bile acid including CA (cholic acid), DCA (deoxycholic acid), TCA (taurine-conjugated cholic acid), TUDCA and LCA were measured as previously described (Qi et al., 2019). After prepared the plasma and ileum samples, bile acid were measured in Ultra Liquid Chromatography 100 system coupled to an AB 5600 TripleTOF system (AB SCIEX), with an XBridge Peptide BEH C18 column (2.13100 mm, 1.7 mm; Waters Corp.). The column temperature was 40 _C, the flow rate was 0.4 mL/min, and the injection volume was 5 mL. The mobile phase solvent is a mixture of 0.1% (v/v) formic acid, 10 mM acetic acid-amine in water (phase A) and 0.1% formic acid in 80% (v/v) methanol and 20% (v/v) acetonitrile (phase B).

*F. nucleatum* ATCC25586 was grown anaerobically (80% N2, 10% CO2 and 10% H2) in agar plate added with 1 μg/mL menadione, 10 μg/mL hemin acquired from Sigma, and 5% sheep blood. The optical density at 600 nm of *F. nucleatum,* was adjusted to 0.06–0.07. The concentration of 20 μg/mL Nal-P-113 was then added to the respective bacterial cultures. Cultures without an antibacterial agent were considered as control group. The plates were incubated for 48 h continuously, a microplate reader was used to analyse the optical density at 600 nm. The tests were performed more than three times.

### 5.4 H&E immunohistochemistry (IHC) and IF staining

Intestine, liver tissues were embedded in paraffin for staining with H&E according to previous reports (Wu et al., 2021). For IHC, the paraffin sections were placed in citrate buffer for antigen retrieval and blocked in 3% H2O2. After incubation with the appropriate primary antibody and secondary antibody, the paraffin sections were blocked with DAB chromogen solution and HRP substrate solution. For IF staining, tissues were fixed with 4% paraformaldehyde at room temperature and permeabilized with 0.05% Triton X-100. Then, the tissues were stained with primary antibodies against the following proteins overnight at 4 °C: Occludin, ZO-1, E-cadherin, N-cadherin. After washing with PBS 3 times, the cells were stained with secondary antibodies. The nuclei were stained with DAPI (Sigma). Images were acquired using a fluorescence confocal microscope (Nikon TI2-E, Crest Optics, X-Light V3, Italy).

### 5.5 Electron microscopy for structural analysis of the gut, and liver

TEM analysis was performed after collection of the gut, and liver tissues by Shiyanjia Lab (www.shiyanjia.com). The tissues were split and treated in a cold fixative solution composed of 2.5% glutaraldehyde at 4 °C for 4 h. After washing with PBS, the specimens were post-fixed in 1% OsO4 at 4 °C for 1 h and washed again with PBS. A graded series of ethanol solutions was used for further dehydration, and the specimens were transferred to be incubated. TEM was performed with a JEM-2100F at 80 kV, and images were acquired using a side-inserted BioScan camera.

### 5.6 Western blot analysis and quantitative real-time PCR (qRT-PCR)

Protein and mRNA expression was assessed using Western blotting and qRT-PCR methods. For Western blotting, proteins from gut tissue, liver tissue, hippocampal tissue and BAT were extracted using protein extraction reagent (Thermo Scientific, 78501). The total proteins were separated by SDS-polyacrylamide gel electrophoresis (SDS-PAGE) and transferred to a polyvinylidene fluoride(PVDF) membrane using a wet transfer apparatus (Bio-Rad PowerPac^TM^Basic, Bio-Rad Mini-Protean Tetra System 10025025 Rev A 12–06250312) as described in our previous reports (Huang et al., 2020b, 2020c). ImageJ was used for band densitometry analysis. For RNA extraction from frozen tissues, TRIzol reagent (Jingcai Bio., Xi'an, Shanxi, China) was used. The relative expression of mRNA was quantified using SYBR Green dye (TB Green Premix Ex Taq II). Specific primers were designed by Green Pharma, Shanghai, China. qRT-PCR was performed with the following program: 95 °C for 10 min, 95 °C for 15s, and 60 °C for 1 min in 40 cycles. The program was performed in a CFX96 Touch apparatus (Bio-Rad). The relative expression was calculated using the 2-ΔΔCT method.

### 5.7 16S rRNA microbiome sequencing

Faecal samples were collected based on the experimental design. The faecal samples were shipped to BioTREE Company, Shanghai, China, for detection using an Illumina NovaSeq. Total cellular DNA was extracted with an E.Z.N.A. Stool DNA Kit (Omega) based on the manufacturer's instructions. The bacterial hypervariable V3–V4 region of 16S rRNA was amplified using the primers 341_F:5′-CCTACGGGNGGCWGCAG-3′ and 802_R:5′-TACNVGGGTATCTAATCC-3′ according to our previously published study (Liu et al., 2019). The library, which was constructed using a TruSeq® kit, was quantified with a Qubit instrument and qRT-PCR. After the library was qualified, a NovaSeq 6000platform was used for sequencing. According to the barcode sequences and the PCR amplification primer sequences, the data for the different samples were separated. After the barcode and primer sequences were removed, FLASH (v1.2.7) was used (http://ccb.jhu.edu/software/FLASH/). The reads in each sample were spliced, and the spliced sequence was considered the raw tag. The raw tags obtained by splicing were filtered strictly to obtain high-quality tags. The QIIMEpipeline (v1.9.1, http://qiime.org/scripts/split_libraries_) was used for tag quality control with FASTQ files. Briefly, for tag trimming, raw tags with continuous low quality scores (default quality threshold: ≤19) were first cut from the first low-quality base site when the base number reached the set length (default length value: 3). Next, for tag length filtering, tags with continuous high-quality bases with lengths less than 75% of the tag length were further filtered out from the tags that remained after trimming. After the above processing steps, chimaeric sequences were removed from the tags. The tag sequences were compared to a species annotation database to detect chimerism (https://github.com/torognes/vsearch/), and the chimaeric sequenceswere removed. Finally, the clean tags were obtained. The UniFrac distance was calculated with QIIME software (version 1.9.1), and an unweighted pair group method with arithmetic mean (UPGMA) sample clustering tree was constructed. Principal component analysis (PCA), principal coordinate analysis (PCoA) and non-metric multi-dimensional scaling (NMDS) analysis were performed with R software (version 2.15.3). PCA was performed with R software's ADE4 package and ggplot2 package; PCoA analysis was performed with R software's WGCNA, stats and ggplot2 packages; and NMDS analysis was performed with R software's vegan package. R software was used to analyse the differences between groups for beta diversity indices, and parametric tests and nonparametric tests were carried out. If there were only two groups, t-tests and Wilcoxon tests were used. If there were more than two groups, Tukey's test and Wilcoxon test of the agricolae package were used.

### 5.8 Untargeted metabolomics

Faecal samples were collected after mice were killed and stored at −80 °C until analysis. Analysis was carried out using ultrahigh-performance liquid tandem chromatography quadrupole time-of-flight mass spectrometry (UHPLC-QTOFMS) by BioTREE Company, Shanghai, China. During detection, both positive ion (POS) mode and negative ion (NEG) mode were used. Then, the missing values were filled with half of the minimum value. Additionally, the internal standard normalization method was employed in this data analysis. The final dataset containing the peak number, sample name and normalized peak area information was imported into the SIMCA15.0.2 software package (Sartorius Stedim Data Analytics AB, Umea, Sweden) for multivariate analysis. The data were scaled and logarithmically transformed to minimize the impacts of both noise and high variance of the variables. After these transformations, PCA, an unsupervised analysis that reduces the dimensionality of the data, was carried out to visualize the distribution and grouping of the samples. The 95% confidence interval in the PCA score plot was used as the threshold to identify potential outliers in the dataset. To visualize group separation and find significantly changed metabolites, supervised orthogonal projections to latent structures-discriminant analysis (OPLS-DA) was applied. Then, a 7-fold cross validation was performed to calculate the values of R2 and Q2. The R2 value indicates how well the variation of a variable is explained, and the Q2 value indicates how well a variable can be predicted. To assess the robustness and predictive ability of the OPLS-DA model, 200 permutations were further conducted. Afterward, the R2 and Q2 intercept values were obtained. Here, the intercept value of Q2 represents the robustness of the model, the risk of over fitting and the reliability of the model (smaller value is better). Furthermore, the variable importance in the projection (VIP) value of the first principal component in OPLS-DA was obtained. This value summarizes the contribution of each variable to the model. The metabolites with VIP>1 and p < 0.05 (Student's t-test) were considered significantly changed metabolites. In addition, commercial databases, including the KEGG (http://www.genome.jp/kegg/) and Metabo Analyst (http://www.metaboanalyst.ca/) databases, were used for pathway enrichment analysis.

### 5.9 Statistical analysis

Gut microbiota abundance, relative metabolite levels, relative protein expression and mRNA expression are presented as the means±SDs. Significant differences between mean results were analysed by Student's t-test and one-way ANOVA, but the results of the antibiotic, IA and probiotic intervention experiments were analysed by two-way ANOVA. The statistical figures were drawn and multiple comparisons via Tukey's test were carried out with GraphPad Prism 8.0 software. ImageJ was used for band densitometry analysis. A value of p < 0.05 was considered to indicate a significant difference between means.

## Ethical approval and consent to participate

The study was approved by the Animal Care and Use Committee at the Central South University.

## Consent for publication

N/A.

## Availability of supporting data

N/A.

## Funding

This study was supported by grants from the 10.13039/501100001809National Natural Science Foundation of China (Grant Nos. 82073486, U1803124), Key Research and Development (R&D) Plan of Hunan Province (Grant Nos.2021SK2026), Natural Science Foundation of Hunan Province (2019JJ40397), and Scientific research project of Hunan Health Committee(202112010058), and the Graduate Research Innovation Program of Central South University (No.2021zzts0325,2021zzts0966,2021zzts0970).

## CRediT authorship contribution statement

**Huiji Pan:** Investigation. **Meiling Zhou:** Investigation. **Zhao Ju:** Investigation. **Jinhua Luo:** Investigation. **Jing Jin:** Investigation. **Liangfang Shen:** Investigation. **Pingkun Zhou:** critically revised manuscript. **Ruixue Huang:** Conceptualization, Formal analysis, Writing – original draft, Funding acquisition.

## Declaration of competing interest

The authors declare that they have no known competing financial interests or personal relationships that could have appeared to influence the work reported in this paper.

## Data Availability

Data will be made available on request.

## References

[bib1] Blasco-Baque V., Serino M., Vergnes J.N., Riant E., Loubieres P., Arnal J.F., Gourdy P., Sixou M., Burcelin R., Kemoun P. (2012). High-fat diet induces periodontitis in mice through lipopolysaccharides (LPS) receptor signaling: protective action of estrogens. PLoS One.

[bib2] Chalasani N., Younossi Z., Lavine J.E., Charlton M., Cusi K., Rinella M., Harrison S.A., Brunt E.M., Sanyal A.J. (2018). The diagnosis and management of nonalcoholic fatty liver disease: practice guidance from the American Association for the Study of Liver Diseases. Hepatology.

[bib3] Chiang J.Y. (2017). Recent advances in understanding bile acid homeostasis. F1000Res.

[bib4] Chung G.E., Cho E.J., Yoon J.W., Yoo J.J., Chang Y., Cho Y., Park S.H., Han K., Shin D.W., Yu S.J. (2021). Nonalcoholic fatty liver disease increases the risk of diabetes in young adults: a nationwide population-based study in Korea. Metabolism.

[bib5] Cucinotta F.A. (2022). Flying without a net: space radiation cancer risk predictions without a gamma-ray basis. Int. J. Mol. Sci..

[bib6] Cuiju W., Shibiao S., Ying T., Rongzong L., Haijuan X., Huifeng C., Tianjian W. (2020). IL-2 and IL-2R gene polymorphisms and immune function in people residing in areas with high background radiation, Yangjiang, China. Int. J. Radiat. Biol..

[bib7] Dai X., Huang R., Hu S., Zhou Y., Sun X., Gui P., Yu Z., Zhou P. (2020). A novel miR-0308-3p revealed by miRNA-seq of HBV-positive hepatocellular carcinoma suppresses cell proliferation and promotes G1/S arrest by targeting double CDK6/Cyclin D1 genes. Cell Biosci..

[bib8] de Vathaire F., El-Fayech C., Ben Ayed F.F., Haddy N., Guibout C., Winter D., Thomas-Teinturier C., Veres C., Jackson A., Pacquement H., Schlumberger M., Hawkins M., Diallo I., Oberlin O. (2012). Radiation dose to the pancreas and risk of diabetes mellitus in childhood cancer survivors: a retrospective cohort study. Lancet Oncol..

[bib9] Dongiovanni P., Meroni M., Baselli G.A., Bassani G.A., Rametta R., Pietrelli A., Maggioni M., Facciotti F., Trunzo V., Badiali S., Fargion S., Gatti S., Valenti L. (2017). Insulin resistance promotes Lysyl Oxidase like 2 induction and fibrosis accumulation in non-alcoholic fatty liver disease. Clin. Sci. (Lond.).

[bib10] Fazel Y., Koenig A.B., Sayiner M., Goodman Z.D., Younossi Z.M. (2016). Epidemiology and natural history of non-alcoholic fatty liver disease. Metabolism.

[bib11] Guzior D.V., Quinn R.A. (2021). Review: microbial transformations of human bile acids. Microbiome.

[bib12] Hild B., Heinzow H.S., Schmidt H.H., Maschmeier M. (2021). Bile acids in control of the gut-liver-Axis. Z. Gastroenterol..

[bib13] Honkoop P., de Man R.A., Scholte H.R., Zondervan P.E., Van Den Berg J.W., Rademakers L.H., Schalm S.W. (1997). Effect of lamivudine on morphology and function of mitochondria in patients with chronic hepatitis B. Hepatology.

[bib14] Huang R.X., Zhou P.K. (2020). DNA damage response signaling pathways and targets for radiotherapy sensitization in cancer. Signal Transduct. Targeted Ther..

[bib15] Huang R., Zhou P.K. (2021). DNA damage repair: historical perspectives, mechanistic pathways and clinical translation for targeted cancer therapy. Signal Transduct. Targeted Ther..

[bib16] Huang R., Ju Z., Zhou P.K. (2020). A gut dysbiotic microbiota-based hypothesis of human-to-human transmission of non-communicable diseases. Sci. Total Environ..

[bib17] Huang R., Gao S., Han Y., Ning H., Zhou Y., Guan H., Liu X., Yan S., Zhou P.K. (2020). BECN1 promotes radiation-induced G2/M arrest through regulation CDK1 activity: a potential role for autophagy in G2/M checkpoint. Cell Death Dis..

[bib18] Huang R., Liu X., Li H., Zhou Y., Zhou P.K. (2020). Integrated analysis of transcriptomic and metabolomic profiling reveal the p53 associated pathways underlying the response to ionizing radiation in HBE cells. Cell Biosci..

[bib19] Huang X., Maguire O.A., Walker J.M., Jiang C.S., Carroll T.S., Luo J.D., Tonorezos E., Friedman D.N., Cohen P. (2021). Therapeutic radiation exposure of the abdomen during childhood induces chronic adipose tissue dysfunction. JCI Insight.

[bib20] Huda M.N., Kim M., Bennett B.J. (2021). Modulating the microbiota as a therapeutic intervention for type 2 diabetes. Front. Endocrinol..

[bib21] Irving B.A., Wood G.C., Bennotti P.N., Babu E., Deshpande A., Lent M.R., Petrick A., Gabrielsen J., Strodel W., Gerhard G.S., Still C.D., Ganapathy V., Rolston D.D. (2016). Nutrient transporter expression in the jejunum in relation to body mass index in patients undergoing bariatric surgery. Nutrients.

[bib22] Ju Z., Ren G.F., Zhou M.L., Jing J., Xiang J., Liu X.D., Huang R.X., Zhou P.K. (2020). Exposure to a combination of silica nanoparticles and low-dose radiation aggravates lung fibrosis in mice via gut microbiota modulation. Environ. Sci-Nano..

[bib23] Kim J., Jung Y. (2017). Radiation-induced liver disease: current understanding and future perspectives. Exp. Mol. Med..

[bib24] Klindt C., Reich M., Hellwig B., Stindt J., Rahnenfuhrer J., Hengstler J.G., Kohrer K., Schoonjans K., Haussinger D., Keitel V. (2019). The G protein-coupled bile acid receptor TGR5 (Gpbar1) modulates endothelin-1 signaling in liver. Cells.

[bib25] Kwan S.Y., Jiao J., Qi J., Wang Y., Wei P., McCormick J.B., Fisher-Hoch S.P., Beretta L. (2020). Bile acid changes associated with liver fibrosis and steatosis in the Mexican-American population of South Texas. Hepatol. Commun..

[bib26] Kwong E., Li Y., Hylemon P.B., Zhou H. (2015). Bile acids and sphingosine-1-phosphate receptor 2 in hepatic lipid metabolism. Acta Pharm. Sin. B.

[bib27] Li R., Andreu-Sanchez S., Kuipers F., Fu J. (2021). Gut microbiome and bile acids in obesity-related diseases. Best Pract. Res. Clin. Endocrinol. Metabol..

[bib28] Lin R., Wu D., Wu F.J., Meng Y., Zhang J.H., Wang X.G., Jia L.H. (2019). Non-alcoholic fatty liver disease induced by perinatal exposure to bisphenol a is associated with activated mTOR and TLR4/NF-kappaB signaling pathways in offspring rats. Front. Endocrinol..

[bib29] Liu X., Zhou Y., Wang S., Guan H., Hu S., Huang R., Zhou P. (2019). Impact of low-dose ionising radiation on the composition of the gut microbiota of mice. Toxicol. Sci..

[bib30] Luo M., An R., Fu J., Wan S., Zhu W., Wang L., Dong Z. (2021). Comparative analysis of the gut microbiota in bighead carp under different culture patterns. J. Appl. Microbiol..

[bib31] Ma C., Han M., Heinrich B., Fu Q., Zhang Q., Sandhu M., Agdashian D., Terabe M., Berzofsky J.A., Fako V., Ritz T., Longerich T., Theriot C.M., McCulloch J.A., Roy S., Yuan W., Thovarai V., Sen S.K., Ruchirawat M., Korangy F., Wang X.W., Trinchieri G., Greten T.F. (2018). Gut microbiome-mediated bile acid metabolism regulates liver cancer via NKT cells. Science.

[bib32] Matsui M., Fukunishi S., Nakano T., Ueno T., Higuchi K., Asai A. (2021). Ileal bile acid transporter inhibitor improves hepatic steatosis by ameliorating gut microbiota dysbiosis in NAFLD model mice. mBio.

[bib33] Mayneris-Perxachs J., Cardellini M., Hoyles L., Latorre J., Davato F., Moreno-Navarrete J.M., Arnoriaga-Rodriguez M., Serino M., Abbott J., Barton R.H., Puig J., Fernandez-Real X., Ricart W., Tomlinson C., Woodbridge M., Gentileschi P., Butcher S.A., Holmes E., Nicholson J.K., Perez-Brocal V., Moya A., Clain D.M., Burcelin R., Dumas M.E., Federici M., Fernandez-Real J.M. (2021). Iron status influences non-alcoholic fatty liver disease in obesity through the gut microbiome. Microbiome.

[bib34] McGlinchey A., Sinioja T., Lamichhane S., Sen P., Bodin J., Siljander H., Dickens A.M., Geng D., Carlsson C., Duberg D., Ilonen J., Virtanen S.M., Dirven H., Berntsen H.F., Zimmer K., Nygaard U.C., Oresic M., Knip M., Hyotylainen T. (2020). Prenatal exposure to perfluoroalkyl substances modulates neonatal serum phospholipids, increasing risk of type 1 diabetes. Environ. Int..

[bib35] Meijles D.N., Fuller S.J., Cull J.J., Alharbi H.O., Cooper S.T.E., Sugden P.H., Clerk A. (2021). The insulin receptor family and protein kinase B (Akt) are activated in the heart by alkaline pH and alpha1-adrenergic receptors. Biochem. J..

[bib36] Milhem A., Abu Toamih-Atamni H.J., Karkar L., Houri-Haddad Y., Iraqi F.A. (2021). Studying host genetic background effects on multimorbidity of intestinal cancer development, type 2 diabetes and obesity in response to oral bacterial infection and high-fat diet using the collaborative cross (CC) lines. Anim. Model Exp. Med..

[bib37] Moore M.P., Cunningham R.P., Dashek R.J., Mucinski J.M., Rector R.S. (2020). A fad too far? Dietary strategies for the prevention and treatment of NAFLD. Obesity.

[bib38] Mortazavi S.A., Mortazavi G., Mortazavi S.M. (2016). Comments on Meo et al. Association of Exposure to Radio-Frequency Electromagnetic Field Radiation (RF-EMFR) Generated by Mobile Phone Base Stations with Glycated Hemoglobin (HbA1c) and Risk of Type 2 Diabetes Mellitus. Int. J. Environ. Res. Publ. Health.

[bib39] Mu H.N., Zhou Q., Yang R.Y., Tang W.Q., Li H.X., Wang S.M., Li J., Chen W.X., Dong J. (2021). Caffeic acid prevents non-alcoholic fatty liver disease induced by a high-fat diet through gut microbiota modulation in mice. Food Res. Int..

[bib40] Nan Y., An J., Bao J., Chen H., Chen Y., Ding H., Dou X., Duan Z., Fan J., Gao Y., Han T., Han Y., Hu P., Huang Y., Huang Y., Jia J., Jiang J., Jiang Y., Li J., Li J., Li R., Li S., Li W., Li Y., Lin S., Liu J., Liu S., Lu L., Lu Q., Luo X., Ma X., Rao H., Ren H., Ren W., Shang J., Shi L., Su M., Wang B., Wang R., Wei L., Wen Z., Wu B., Wu J., Xin S., Xing H., Xu J., Yan M., Yang J., Yang J., Yang L., Yang Y., Yu Y., Zhang L., Zhang L., Zhang X., Zhang Y., Zhang Y., Zhao J., Zhao S., Zheng H., Zhou Y., Zhou Y., Zhuang H., Zuo W., Xu X., Qiao L. (2021). The Chinese Society of Hepatology position statement on the redefinition of fatty liver disease. J. Hepatol..

[bib41] Nobakht H., Mahmoudi T., Sabzikarian M., Tabaeian S.P., Rezamand G., Asadi A., Farahani H., Dabiri R., Mansour-Ghanaei F., Maleki I., Zali M.R. (2020). Insulin and insulin receptor gene polymorphisms and susceptibility to nonalcoholic fatty liver disease. Arq. Gastroenterol..

[bib42] Nylander V., Ingerslev L.R., Andersen E., Fabre O., Garde C., Rasmussen M., Citirikkaya K., Baek J., Christensen G.L., Aznar M., Specht L., Simar D., Barres R. (2016). Ionizing radiation potentiates high-fat diet-induced insulin resistance and reprograms skeletal muscle and adipose progenitor cells. Diabetes.

[bib43] Qi X., Yun C., Sun L., Xia J., Wu Q., Wang Y., Wang L., Zhang Y., Liang X., Wang L., Gonzalez F.J., Patterson A.D., Liu H., Mu L., Zhou Z., Zhao Y., Li R., Liu P., Zhong C., Pang Y., Jiang C., Qiao J. (2019). Gut microbiota-bile acid-interleukin-22 axis orchestrates polycystic ovary syndrome. Nat. Med..

[bib44] Radun R., Trauner M. (2021). Role of FXR in bile acid and metabolic homeostasis in NASH: pathogenetic concepts and therapeutic opportunities. Semin. Liver Dis..

[bib45] Rozos G., Voidarou C., Stavropoulou E., Skoufos I., Tzora A., Alexopoulos A., Bezirtzoglou E. (2018). Biodiversity and microbial resistance of lactobacilli isolated from the traditional Greek cheese kopanisti. Front. Microbiol..

[bib46] Sabol R.A., Villela V.A., Denys A., Freeman B.T., Hartono A.B., Wise R.M., Harrison M.A.A., Sandler M.B., Hossain F., Miele L., Bunnell B.A. (2020). Obesity-altered adipose stem cells promote radiation resistance of estrogen receptor positive breast cancer through paracrine signaling. Int. J. Mol. Sci..

[bib47] Shao J.W., Ge T.T., Chen S.Z., Wang G., Yang Q., Huang C.H., Xu L.C., Chen Z. (2021). Role of bile acids in liver diseases mediated by the gut microbiome. World J. Gastroenterol..

[bib48] Sharma A., Shrivastava S., Shukla S. (2021). Oxidative damage in the liver and brain of the rats exposed to frequency-dependent radiofrequency electromagnetic exposure: biochemical and histopathological evidence. Free Radic. Res..

[bib49] Skonieczna-Zydecka K., Grochans E., Maciejewska D., Szkup M., Schneider-Matyka D., Jurczak A., Loniewski I., Kaczmarczyk M., Marlicz W., Czerwinska-Rogowska M., Pelka-Wysiecka J., Dec K., Stachowska E. (2018). Faecal short chain fatty acids profile is changed in polish depressive women. Nutrients.

[bib50] Stokowa-Soltys K., Wojtkowiak K., Jagiello K. (2021). Fusobacterium nucleatum - friend or foe?. J. Inorg. Biochem..

[bib51] Thorby-Lister A., Hogler W., Hodgson K., Crabtree N., Nightingale P., Shaw N., Saraff V. (2018). Cumulative radiation exposure from medical imaging and associated lifetime cancer risk in children with osteogenesis imperfecta. Bone.

[bib52] Vaiserman A., Cuttler J.M., Socol Y. (2021). Low-dose ionizing radiation as a hormetin: experimental observations and therapeutic perspective for age-related disorders. Biogerontology.

[bib53] Wan J., Zhang Y., Yang D., Liang Y., Yang L., Hu S., Liu Z., Qian F., Tian S., Ding Y. (2021). Gastrodin improves nonalcoholic fatty liver disease via activation of the AMPK signaling pathway. Hepatology.

[bib54] Weng Y.J., Gan H.Y., Li X., Huang Y., Li Z.C., Deng H.M., Chen S.Z., Zhou Y., Wang L.S., Han Y.P., Tan Y.F., Song Y.J., Du Z.M., Liu Y.Y., Wang Y., Qin N., Bai Y., Yang R.F., Bi Y.J., Zhi F.C. (2019). Correlation of diet, microbiota and metabolite networks in inflammatory bowel disease. J. Dig. Dis..

[bib55] Wu L., Liu J., Tian X., Groleau R.R., Bull S.D., Li P., Tang B., James T.D. (2021). Fluorescent probe for the imaging of superoxide and peroxynitrite during drug-induced liver injury. Chem. Sci..

[bib56] Yahyapour R., Amini P., Rezapour S., Cheki M., Rezaeyan A., Farhood B., Shabeeb D., Musa A.E., Fallah H., Najafi M. (2018). Radiation-induced inflammation and autoimmune diseases. Mil. Med. Res..

[bib57] Yang P., Liang Y., Luo Y., Li Z., Wen Y., Shen J., Li R., Zheng H., Gu H.F., Xia N. (2019). Liraglutide ameliorates nonalcoholic fatty liver disease in diabetic mice via the IRS2/PI3K/Akt signaling pathway. Diabetes Metab. Syndr. Obes..

[bib58] Yu W., Wang M., Cai L., Jin Y. (1995). Pre-exposure of mice to low dose or low dose rate ionizing radiation reduces chromosome aberrations induced by subsequent exposure to high dose of radiation or mitomycin C. Chin. Med. Sci. J..

[bib59] Zhang F., Zhang Z., Kong D., Zhang X., Chen L., Zhu X., Lu Y., Zheng S. (2014). Tetramethylpyrazine reduces glucose and insulin-induced activation of hepatic stellate cells by inhibiting insulin receptor-mediated PI3K/AKT and ERK pathways. Mol. Cell. Endocrinol..

[bib60] Zhang Y., Ren H., Zheng Y., Yang Q., Li M., Gu H., Hao L. (2021). Exploring the optimal dose of low ionizing radiation to enhance immune function: a rabbit model. J. Int. Med. Res..

[bib61] Zhang J., Zhang L., Xie B., Duan Y., Wang Y., Shen L. (2021). PKCalpha is a potentially useful marker for planning individualized radiotherapy for nasopharyngeal carcinoma. Cancer Manag. Res..

[bib62] Zietek M., Celewicz Z., Kikut J., Szczuko M. (2021). Implications of SCFAs on the parameters of the lipid and hepatic profile in pregnant women. Nutrients.

