# Supplementary material for: Potential role of gut microbiota-LCA-INSR axis in high fat-diet-induced non-alcoholic fatty liver dysfunction: From perspective of radiation variation
Source: Curr Res Food Sci. 2022 Sep 27;5:1685–700. doi: 10.1016/j.crfs.2022.09.022 (PMC9530674; doi:10.1016/j.crfs.2022.09.022)
Supplement: Multimedia component 1 [file mmc1.docx]

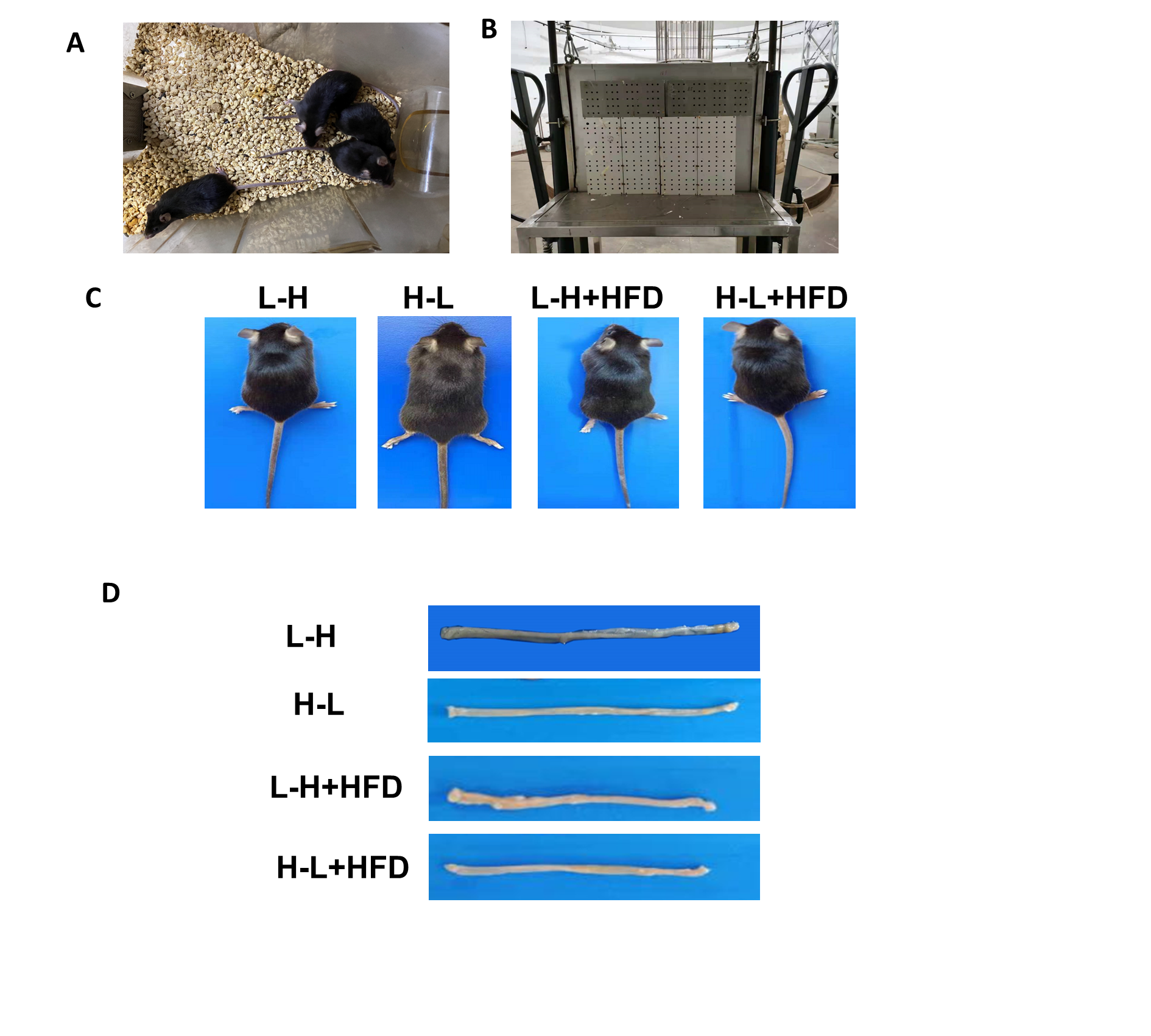


Figure S1. A. mice used in this study. B. Radiation device used for mice radiation exposure. The radiation resource is Co60. C. mice in 4 groups. D. Intestine tissues in 4 groups at the 15^th^ week post various intervention.


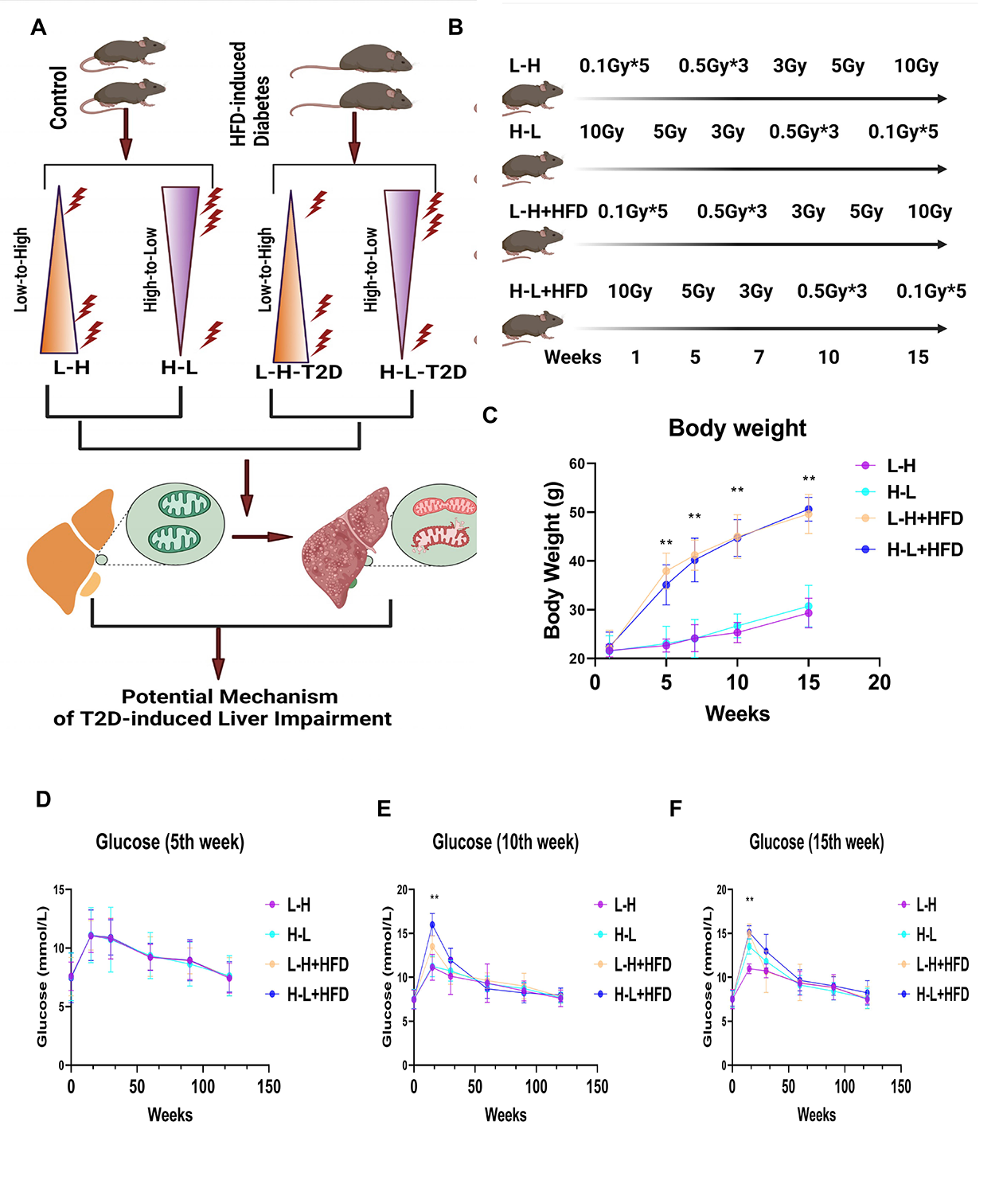


Figure S2. High-fat-diet synergized with repeated exposure to radiation variation triggers stronger glucose dysfunction in mice. A. Experimental protocol for this study. Mice were categorized into 4 group, repeated radiation exposure from initial low dose(0.1Gy five times) to high dose(10Gy) at abdominal region for 15 weeks(L-H group); repeated radiation exposure from initial high dose(10Gy) to low dose(0.1Gy five times) at abdominal region for 15 weeks(H-L group); HFD-fed combined with repeated radiation exposure from initial high dose(10Gy) to low dose(0.1Gy five times) at abdominal region for 15 weeks(L-H+HFD group) and HFD-fed combined with repeated radiation exposure from initial high dose(10Gy) to low dose(0.1Gy five times) at abdominal region for 15 weeks(H-L+HFD group). B. Timeline for the mice subjected to radiation dose variations in 4 groups. C. Body weight alterations of mice during 15 weeks among from for groups. D. Blood glucose levels at indicated time points among L-H, H-L, L-H+HFD and H-L+HFD groups at the 5^th^ week post intervention. E. Blood glucose levels at indicated time points among L-H, H-L, L-H+HFD and H-L+HFD groups at the 10^th^ week post intervention. F. Blood glucose levels at indicated time points among L-H, H-L, L-H+HFD and H-L+HFD groups at the 15^th^ week post intervention. Data are means ± standard deviation (SD). Mann-Whitney test or two-tailed unpaired Student’s t-test were used for statistical analyses. **p*<0.05 indicates significant difference.


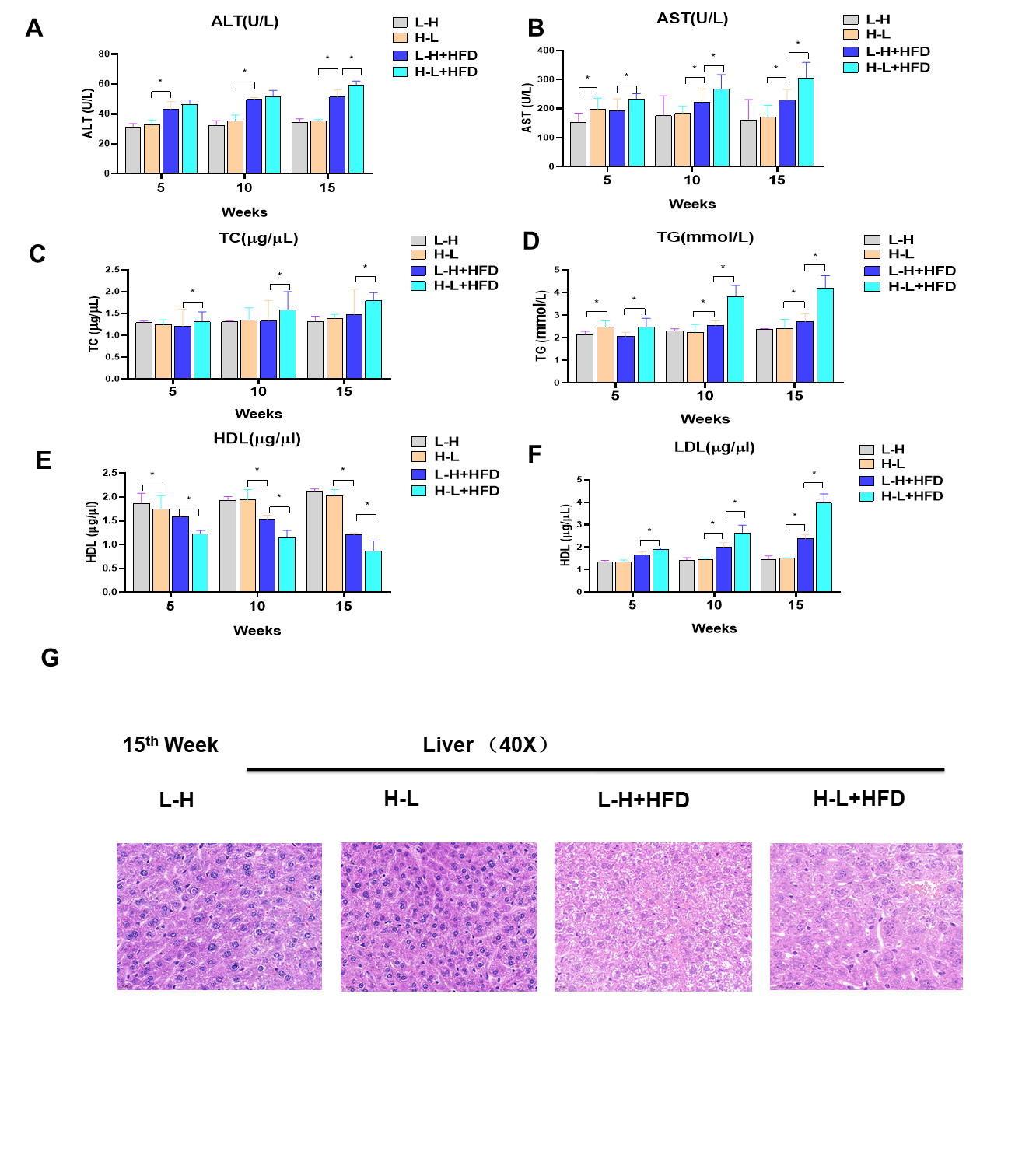


Figure S3. High-fat-diet synergized with repeated exposure to radiation variation triggers stronger liver dysfunction in mice. A. ALT (alanine aminotransferase) levels in mice (n=5 per group). B. AST（glutamic oxaloacetic transaminase）levels in mice (n=5 per group). C. TC（total cholesterol）levels in mice (n=5 per group). D. TG (triglyceride) levels in mice (n=5 per group). E. HDL (high density lipoprotein) levels in mice (n=5 per group). F. LDL (low density lipoprotein) levels in mice (n=5 per group). G. Representative pictures of liver tissues stained with H&E (40X) among 4 groups at 15^th^ week post HFD or radiation intervention. Data are means ± standard deviation (SD). Mann-Whitney test or two-tailed unpaired Student’s t-test were used for statistical analyses. **p*<0.05 indicates significant difference.


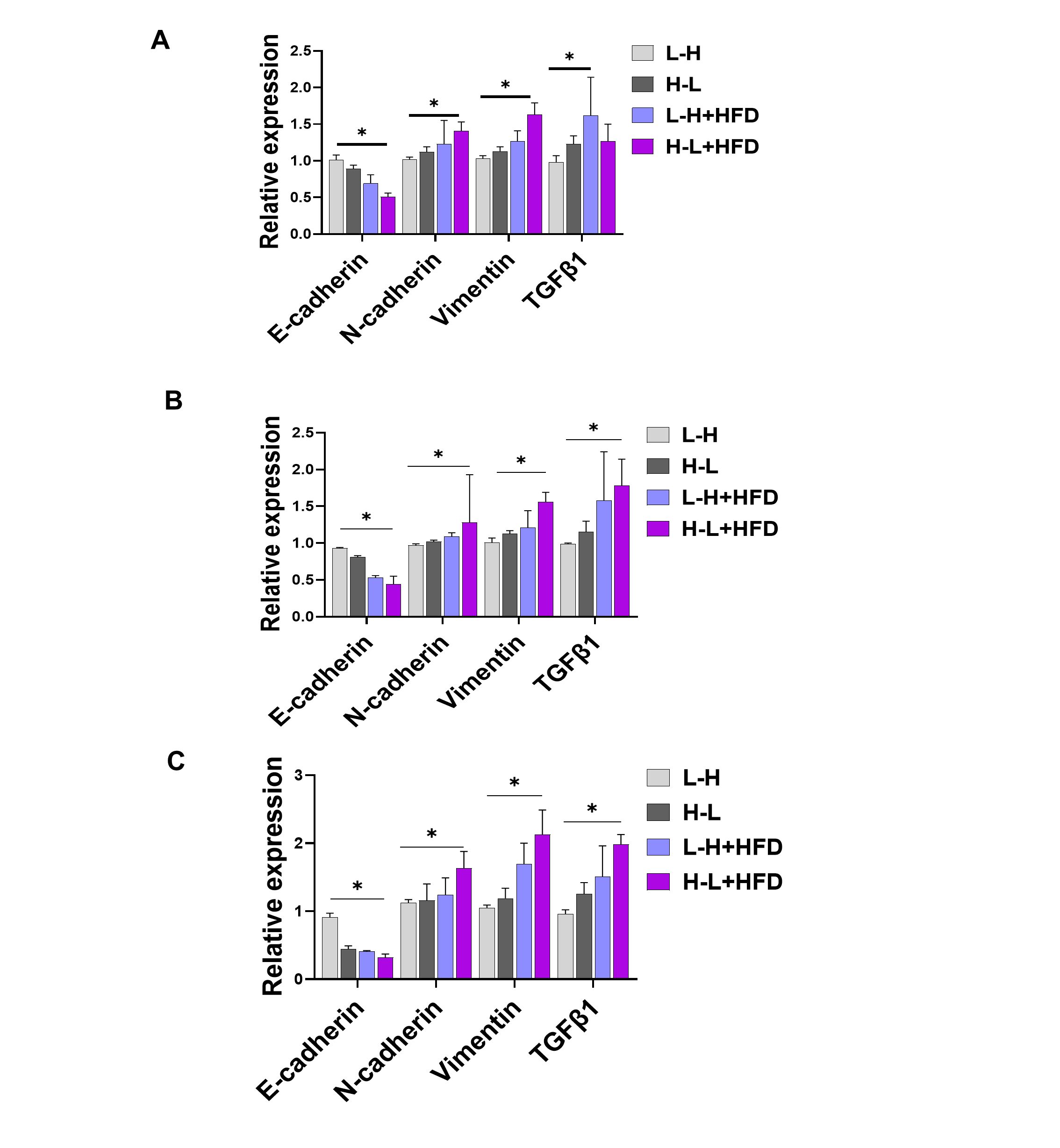


Figure S4. Qualification of EMT-related proteins expression at 5, 10, and 15 weeks among 4 groups. A. Qualification of EMT-related proteins expression at 5 weeks among 4 groups. B. Qualification of EMT-related proteins expression at 10 weeks among 4 groups. C. Qualification of EMT-related proteins expression at 15 weeks among 4 groups.


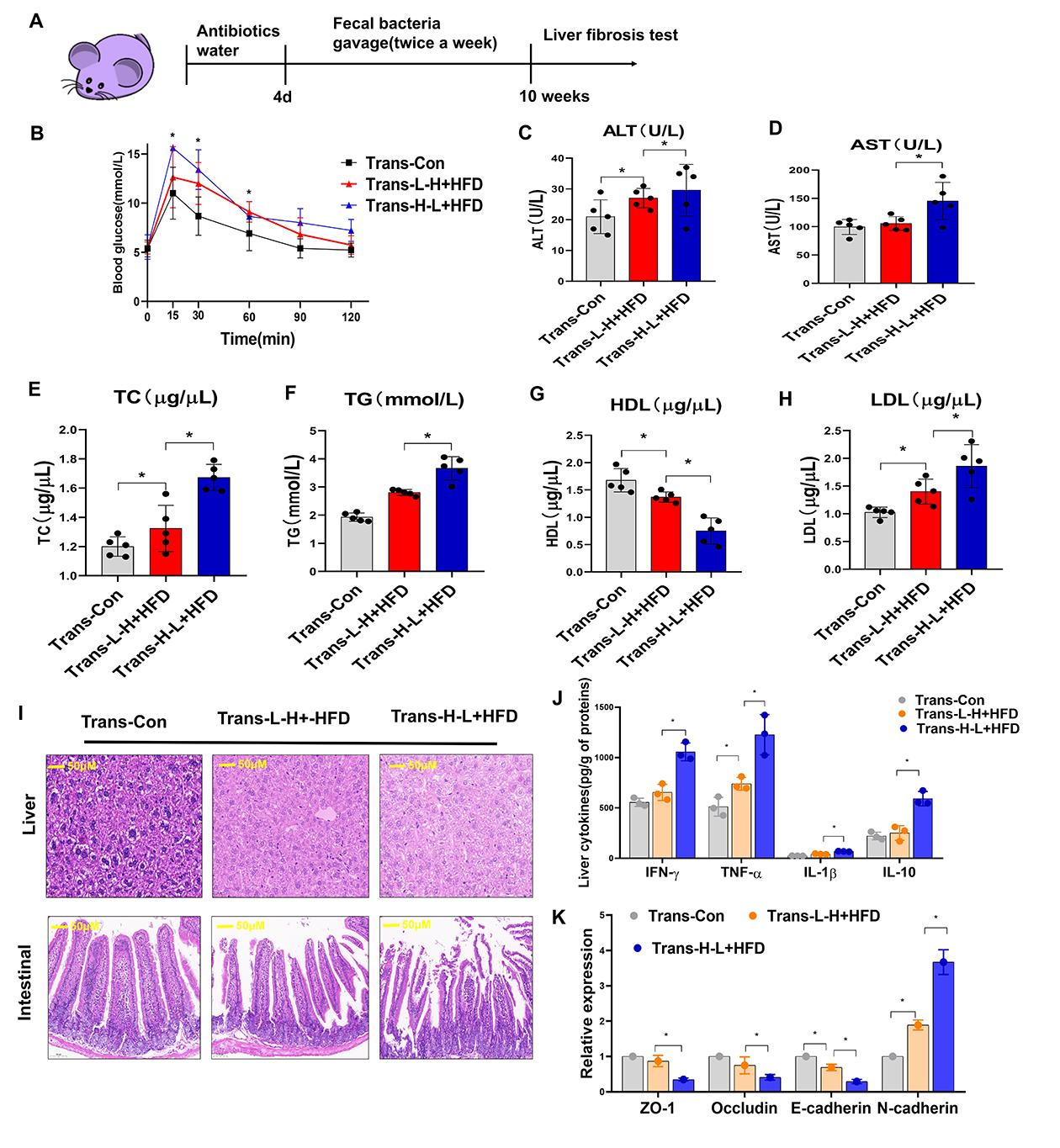


Figure S5. Effects of L-H+HFD, H-L+HFD fecal microbiota transplantation disrupted liver function. A. Timeline of the mice transplanted with stool suspensions from healthy controls, L-H+HFD cases and H-L+HFD cases were defined as Trans-Con, Trans-L-H+HFD and Trans-H-L+HFD, respectively. After treatment with antibiotics in drinking water for 4 days, the mice were gavaged with stool suspensions twice per week. B. Glucose levels (n=5 per group). C. ALT (n=5 per group). D. ITT AST (n=5 per group). E. TC level (n=5 per group). F. TG level (n=5 per group). G. HDL level (n=5 per group). H. LDL level (n=5 per group). I. H&E staining of representative liver and intestinal, scale bar, 50μm. J. Liver cytokines levels, IFN-γ, TNF-a, IL-1β, IL-10 in 3 groups. K. Relative expression of intestinal ZO-1, Occludin, E-cadherin and N-cadherin among 3 groups. Data are means ± standard deviation (SD). Mann-Whitney test or two-tailed unpaired Student’s t-test were used for statistical analyses**p*<0.05 indicates significant difference.


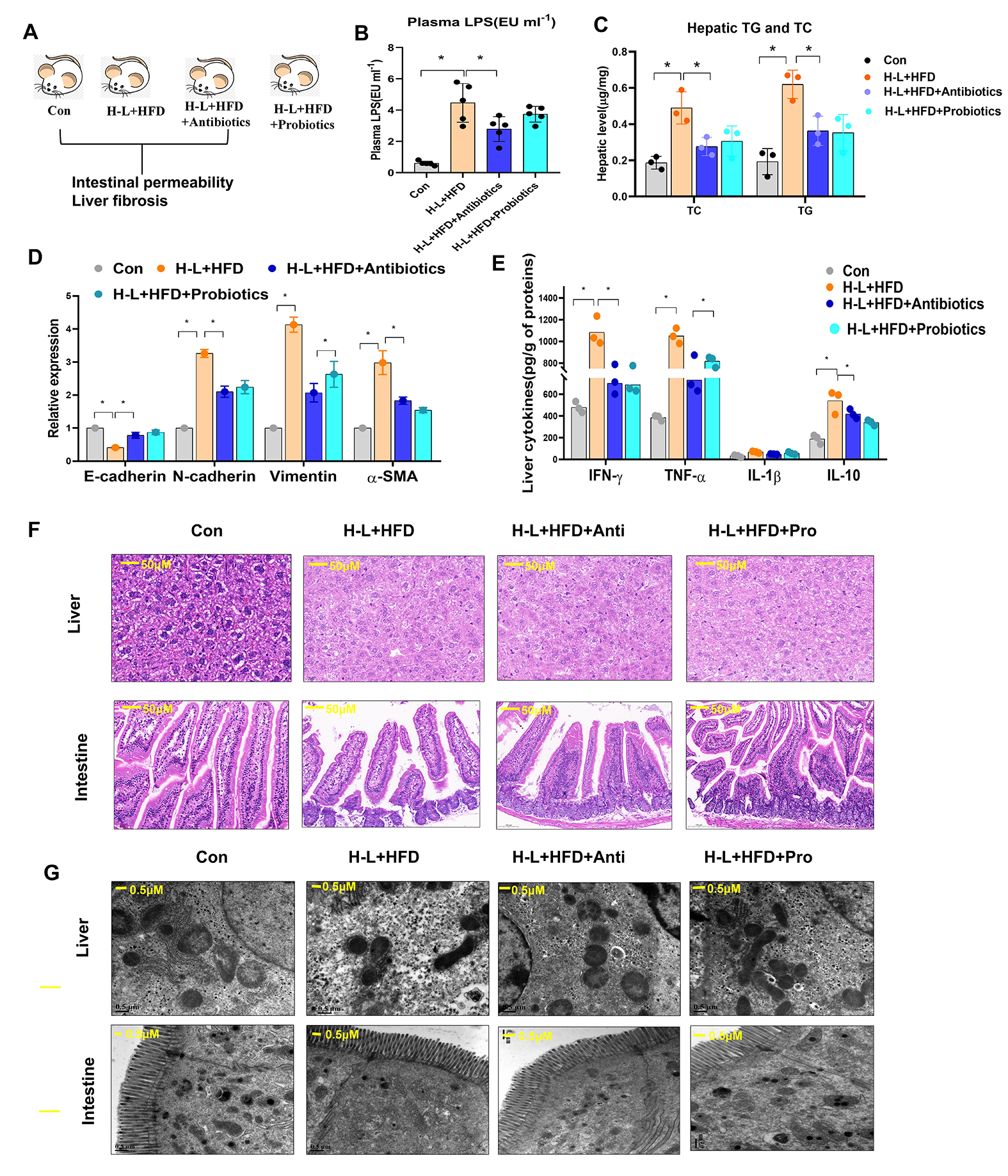


Figure S6. Antibiotics intervention reverse the H-L+HFD-induced intestinal impairments and liver fibrosis. Mice were divided into four groups, con, H-L+HFD, H-L+HFD+Antibiotics (antibiotics cocktail) and H-L+HFD+Probiotics (Lactobacillus rhamnosus and Lactobacillus reuteri complex). A. Timeline of mice subjected to H-L+HFD, H-L+HFD+Antibiotics and H-L+HFD+Probiotics. B. Plasma LPS level. C. hepatic TG and TC levels. D. Relative expression of E-cadherin, N-cadherin, Vimentin and a-SMA. E. Liver cytokines of IFN-γ, TNF-a, IL-1β, IL-10. F. H&E staining of representative liver and intestinal, scale bar, 50μM. G. Representative electron microscopy images of liver and intestine tissues among 4 groups, scale bar: 0.5μm. Data are means ± standard deviation (SD). Mann-Whitney test or two-tailed unpaired Student’s t-test were used for statistical analyses**p*<0.05 indicates significant difference.


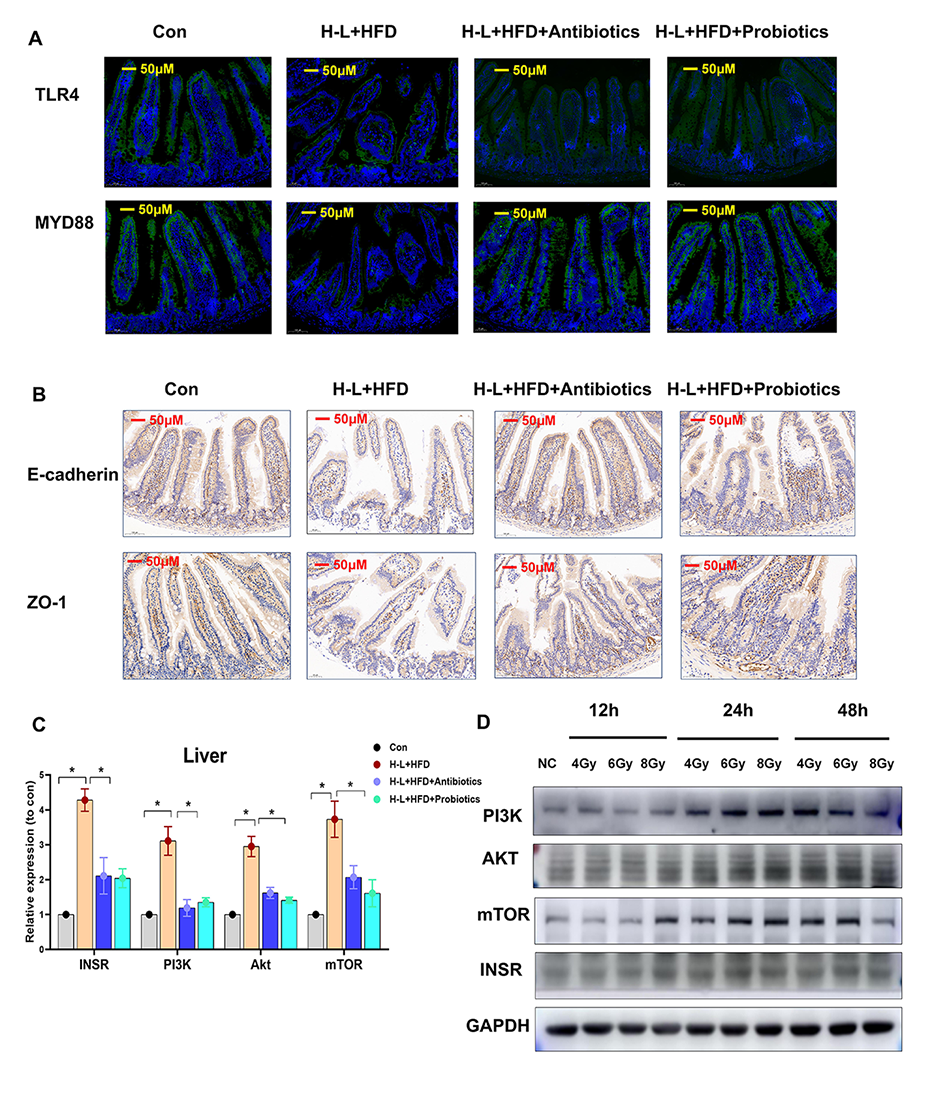


Figure S7. Antibiotics intervention reverse the H-L+HFD-induced -induced insulin resistance. A. Immunofluorescence detection for TLR4 and MYD88 expression in mice treated with H-L+HFD, H-L+HFD+Antibiotics and H-L+HFD+Probiotics, scale bar: 100μM. B. Immunohistochemistry detection for E-cadherin and ZO-1 in intestinal tissues. Scale bar:50μM. C. Relative expression of liver INSR, PI3K, Akt and mTOR. D. Western blots of liver insulin singling pathway-related proteins including PI3K, Akt, mTOR and INSR were conducted (n=3 per group). GAPDH subjected to the control. Data are means ± standard deviation (SD). Mann-Whitney test or two-tailed unpaired Student’s t-test were used for statistical analyses. **p*<0.05 indicates significant difference.

Supplementary table 1 | Comparative alterations of gut microbiota relative abundance at phylum level among groups at 15 weeks in mice

| **Up (H-L+HFD vs. L-H+HFD)** | | | | | | | **Down(H-L+HFD vs. L-H+HFD)** | | | | | | |
| --- | --- | --- | --- | --- | --- | --- | --- | --- | --- | --- | --- | --- | --- |
| **Relative abundance(phylum)** | | |  |  | | | **Relative abundance(phylum)** | | |  |  | | |
| **Taxonomy** | **L-H** | **H-L** | **L-H+HFD** | **H-L+HFD** |  |  | **Taxonomy** | **L-H** | **H-L** | **L-H+HFD** | **H-L+HFD** |  |  |
| Actinobacteria | 0.028222 | 0.009282 | 0.01324 | 0.08123 |  |  | Akkermansia muciniphila | 0.03913 | 0.01245 | 0.02013 | 0.005142 |  |  |
| Weissella | 0.601863 | 0.638998 | 0.02134 | 0.2178 |  |  | Prevotellaceae | 0.418477 | 0.25372 | 0.01962 | 0.003743 |  |  |
| Chloroflexi | 0.000005 | 0.000594 | 0.01099 | 0.1964 |  |  | Tannerellaceae | 0.4642 | 0.1012 | 0.03694 | 0.03011 |  |  |
| Cyanobacteria | 0.001044 | 0.001139 | 0.0289 | 0.04690 |  |  | Rikenellacear_RC9 | 0.003318 | 0.003089 | 0.02345 | 0.006123 |  |  |
| Deferribacteres | 0.000055 | 0.000148 | 0.01159 | 0.03459 |  |  | Bacteroidetes | 0.036321 | 0.004378 | 0.042641 | 0.000974 |  |  |
| Epsilonbacteraeota | 0.038213 | 0.008091 | 0.008198 | 0.021309 |  |  | Anaeroplasma | 0.00307 | 0.00212 | 0.00847 | 0.000814 |  |  |
| Firmicutes | 0.292553 | 0.263711 | 0.01459 | 0.022065 |  |  | Marvinbryantia | 0.001182 | 0.0001234 | 0.008591 | 0.0005123 |  |  |
| Fusobacteria | 0.000657 | 0.00014 | 0.00312 | 0.18639 |  |  | Parvibacter | 0.000589 | 0.000501 | 0.000912 | 0.00047 |  |  |
| Patescibacteria | 0.008084 | 0.004736 | 0.004567 | 0.006790 |  |  | Acetatifactor | 0.000213 | 0.00056 | 0.00512 | 0.000917 |  |  |
| Proteobacteria | 0.024305 | 0.020021 | 0.005148 | 0.0067 |  |  | Candidatus_Stoquefichus | 0.000199 | 0.00087 | 0.000228 | 0.000149 |  |  |
| Tenericutes | 0.001837 | 0.001486 | 0.000377 | 0.00971 |  |  | Harryflintia | 0.001112 | 0.000031 | 0.002789 | 0.0003557 |  |  |
| Verrucomicrobia | 0.003163 | 0.051653 | 0.002743 | 0.005129 |  |  | Butyricococcus | 0.002198 | 0.001249 | 0.001203 | 0.001025 |  |  |
| Helicobacteraceae | 0.004129 | 0.088321 | 0.004675 | 0.09123 |  |  | Clostridiaceae | 0.007082 | 0.002954 | 0.004203 | 0.003325 |  |  |

Supplementary table 2 | Comparative alterations of gut microbiota relative abundance at species level among groups at 15 weeks in mice

| **Up (H-L+HFD vs. L-H+HFD)** | | | | | | | **Down(H-L+HFD vs. L-H+HFD)** | | | | | | |
| --- | --- | --- | --- | --- | --- | --- | --- | --- | --- | --- | --- | --- | --- |
| **Relative abundance(species)** | | |  |  | | | **Relative abundance(species)** | | |  |  | | |
| **Taxonomy** | **L-H** | **H-L** | **L-H+HFD** | **H-L+HFD** |  |  | **Taxonomy** | **L-H** | **H-L** | **L-H+HFD** | **H-L+HFD** |  |  |
| Bacteroides_gallinaceum | 0.000002 | 0.0000001 | 0.00001 | 0.000258 |  |  | Bacteroides_acidifaciens | 0.000019 | 0.000009 | 0.001064 | 0.000953 |  |  |
| Firmicutes_bacterium_ZOR0006 | 0.0000013 | 0.0000087 | 0.000005 | 0.000067 |  |  | Bacteroides_plebeius | 0.000035 | 0.0000042 | 0.000558 | 0.000373 |  |  |
| Lactobacillus_aviarius | 0.00000067 | 0.0000074 | 0.000011 | 0.000139 |  |  | Butyricimonas_synergistica | 0.000068 | 0.000012 | 0.000504 | 0.00034 |  |  |
| Syphacia_muris | 0.0000003 | 0.0000006 | 0.000174 | 0.001515 |  |  | Clostridiales_bacterium_CIEAF_020 | 0.0000504 | 0.0000002 | 0.003196 | 0.000533 |  |  |
| [Clostridium]_spiroforme_DSM_1552 | 0.000001 | 0.000003 | 0.000086 | 0.000135 |  |  | Clostridium_sp | 0.000812 | 0.00021 | 0.000137 | 0.000107 |  |  |
| bacterium_c_Bacteroidia | 0.000003 | 0.0000015 | 0.001596 | 0.002271 |  |  | Lachnospiraceae_bacterium_609 | 0.000004 | 0.0000021 | 0.003951 | 0.000279 |  |  |
| *Fusobacterium nucleatum* | 0.000011 | 0.0000043 | 0.002345 | 0.00983 |  |  | Clostridium_sp | 0.000886 | 0.0000935 | 0.0077 | 0.00092 |  |  |
| Bacteroides_acidifaciens | 0.0000009 | 0.00000004 | 0.00167 | 0.0023 |  |  | Firmicutes_bacterium_ZOR0006 | 0.0000009 | 0.00000004 | 0.00082 | 0.00037 |  |  |
| Bacteroides_plebeius | 0.00002 | 0.0000003 | 0.015 | 0.0032 |  |  | Lactobacillus_aviarius | 0.000108 | 0.000006 | 0.0063 | 0.00082 |  |  |
| Butyricimonas_synergistica | 0.00001 | 0.00002 | 0.0005 | 0.0008 |  |  | Parabacteroides_merdae | 0.000076 | 0.000034 | 0.000312 | 0.000298 |  |  |
| Clostridiales_bacterium_CIEAF_020 | 0.000002 | 0.0000001 | 0.00096 | 0.00832 |  |  |  |  |  |  |  |  |  |
| Lachnospiraceae_bacterium_609 | 0.000002 | 0.0000001 | 0.00067 | 0.0013 |  |  |  |  |  |  |  |  |  |
